# ‘Involve those who are managing these outbreaks’: stakeholders’ perspectives on the barriers and facilitators to the implementation of clinical management guidelines for high-consequence infectious diseases in Uganda—a thematic network analysis

**DOI:** 10.1136/bmjph-2024-001165

**Published:** 2025-02-13

**Authors:** Olive Kabajaasi, Stefan Schilling, Mathias Akugizibwe, Peter W Horby, Peter Hart, Louise Sigfrid, Shevin T Jacob

**Affiliations:** 1 Walimu, Kampala, Uganda; 2 Psychology, Faculty of Health and Life Sciences, University of Exeter, Exeter, UK; 3 Pandemic Sciences Institute, University of Oxford, Oxford, UK; 4 Social Sciences Department, Medical Research Council, Entebbe, Uganda; 5 Wellcome Trust, London, UK; 6 Department of Clinical Sciences, Liverpool School of Tropical Medicine, Liverpool, UK

**Keywords:** Disease Outbreaks, Public Health Practice, education, standards, COVID-19

## Abstract

**Introduction:**

Prior research highlighting the complexity of clinical management guidelines’ (CMGs) implementation during high consequence infectious disease (HCID) outbreaks has suggested that limited access to treatments and equipment and substantial issues regarding availability, inclusivity, quality and applicability hinders the implementation of CMGs in low- and middle-income countries (LMICs). This in-depth case study of Uganda—coincidentally occurring during the 2022 Sudan virus disease outbreak—aimed to explore contextual and supplementary factors which hinder or facilitate CMG development and implementation.

**Methods:**

Between August and December 2022, 43 interviews were conducted with medical personnel, consultant physicians, case managers and Uganda Ministry of Health officials. Interviews were analysed using a thematic network analysis approach to visualise thematic codes in qualitative data and highlight inherent relationships between codes.

**Results:**

Six thematic topics emerged as the main barriers to the implementation of CMGs during HCID outbreaks in Uganda: (1) deficient content and slow updates of CMGs; (2) scarce resources and healthcare disparities; (3) slow dissemination and limited access to guidelines; (4) improvisation of patient care (5) lack of training for healthcare workers (HCWs); and (6) limited pandemic preparedness and response infrastructure. Codes most strongly linked to facilitators and suggestions included: (1) HCW training in CMG implementation; (2) adequate resourcing; (3) involvement of personnel with prior HCID response experience in CMG development and (4) improvements in access to CMGs.

**Conclusions:**

By illustrating linkages to resource constraints, healthcare disparities, and limited surveillance and referral infrastructure, our study displays how insufficient training, patchy dissemination and slow updating exacerbate many of the underlying difficulties for CMG implementation in LMIC contexts. Findings offer valuable insights for LMICs to improve HCID outbreak responses and inform implementation of CMGs in future HCID outbreaks, where evidence is often initially limited. Recommendations to enhance CMG implementation are provided.

**WHAT IS ALREADY KNOWN ON THIS TOPIC**

- Previous research has shown that the quality, scope and inclusivity of clinical management guidelines (CMGs) for high consequence infectious disease (HCID) outbreaks are seldom meeting the standards for CMG development and are often unavailable where needed. Evidence also suggests that the provision of supportive care during HCID outbreaks is inhibited by political, personnel and organisational issues (eg, lack of guidelines, shortage of HCW and camaraderie between them). However, little research to date has addressed the specific factors hindering or supporting the implementation of guidelines, for example, why are healthcare workers not using guidelines—even if they exist.

**WHAT THIS STUDY ADDS**

- Through a qualitative case study involving key informant and in-depth interviews with relevant stakeholders in Uganda, the study provides multilayered insights into the barriers and facilitators for CMG implementation during two recent outbreaks namely Ebola and COVID-19. The study highlights important barriers including factors related to insufficient content and infrequency of updates; lack of material and human resources; limited dissemination rates and access; compromised care quality; lack of available training; and lack of availability of specific infrastructure for pandemic preparedness and response.

**HOW THIS STUDY MIGHT AFFECT RESEARCH, PRACTICE OR POLICY**

- The study provides actionable recommendations to improve future guideline development and dissemination practices and help improve the quality of care for patients not only in Uganda but also for other settings affected by HCIDs. These contextual realities provided by stakeholders regarding design, decision-making and issues related to ensuring relevancy and fostering trust, and ownership and compliance deepen our understanding of how CMGs can be implemented in more applicable and impactful ways in resource-constrained settings during high stake public emergencies.

## Introduction

 The COVID-19 pandemic, which resulted in over 700 million people infected worldwide and over 6 million deaths,^1^ has highlighted the necessity for harmonised clinical management guidelines (CMGs) for supportive care of critically ill patients during high consequence infectious disease (HCID) outbreaks, particularly in the early stages of an outbreak. Recent studies, however, have found that CMGs for various HCIDs are either often unavailable in the regional context where they are needed^2^,^6^ or do not meet the agreed on gold standards for CMG development.^7 8^ Some studies suggest guideline adherence by healthcare staff is often impeded by a range of personnel, policy or organisational issues,^9^,^12^ with work exploring the necessary factors for providing supportive care during HCID outbreaks highlighting lack of guidelines, HCW shortages and camaraderie as main barriers to care.^13 14^ However, little research to date has addressed the barriers or facilitators for the implementation of guidelines, for example, why are healthcare workers (HCWs) not using guidelines—even if they exist.

This in-depth case study of Uganda aims to expand on this literature by identifying both distinct barriers and challenges reported by HCWs involved in HCID response and concrete factors that facilitate and support the successful implementation of guidelines by HCWs. As part of a wider research effort to evaluate clinical management guidelines for high consequence infectious diseases (ESHCID) aimed at (a) assessing gaps in available CMGs providing evidence-based recommendations for HCIDs with epidemic and pandemic potential and (b) identifying facilitators and challenges for CMG implementation in different regional contexts and across different HCIDs, this case study was purposefully selected due to Uganda’s extensive experience with HCID outbreaks.

## Background

In the absence of vaccines or directed treatment for most priority pathogens on the WHO Research and Development (R&D) Blueprint list,^15 16^ early and well monitored supportive care is often the only available treatment. As such, high-quality CMGs defined as ‘systematically developed statements to assist practitioner and patient care decisions about appropriate healthcare for specific clinical circumstances’^17^ are crucial tools for standardising supportive care in a way that optimises outcomes for patients and safety for HCWs. Best practice calls for guidelines to be contextually relevant, accessible for use by front-line clinicians, of good quality and inclusive of vulnerable patient groups.^18 19^ Considering the limited empirical knowledge on clinical management of HCIDs, relevant CMGs need to be responsive to incorporating emerging evidence for dissemination to frontline HCWs.

During the COVID-19 pandemic, the WHO produced ‘living guidelines’—a template for optimising the CMG development process to ensure high-quality, up-to-date, evidence-based guidance—is made available to frontline clinicians in time with updates to existing evidence.^20 21^ Living guidelines, however, demand substantial investment and automated processes requiring provision by large national or supranational institutions, thus making them a difficult to adapt model for CMG development in low- and middle-income countries’ (LMICs) contexts.

Recent studies suggest that multiple factors hinder CMG implementation, including policy, organisational and inter-personal issues, alongside time and resource constraints (eg, financial, human and expertise) and limited HCW awareness of existing guidelines and recommendations.^22 23^ These findings are supported by a series of systematic evidence reviews of existing CMGs for viral haemorrhagic fevers, chikungunya, mpox and severe acute respiratory syndrome, which found similar issues regarding availability, inclusivity, quality and applicability of CMGs,^2^,^6^ particularly in LMICs. Additionally, previous responses to public health emergencies have routinely been characterised by a lack of collaboration and consensus, comprehensiveness and contextual relevance.^78 24^,^26^ For example, during the 2013–2016 West African Ebola virus disease (EVD) outbreak, lack of consensus by response stakeholders for CMGs on several high-priority supportive care interventions hindered the standardisation of best practices.^13 14^

Uganda’s experience of HCID outbreak response and role in the development of supportive care guidance over the last two decades allows for insight into the process and uptake of CMGs. As a result of multiple outbreaks of Filovirus diseases (FVDs) since 2000 (see online supplemental file 8), the Ugandan Ministry of Health (MoH) developed a pocket manual in 2012 that provided guidance for frontline HCWs to provide optimal and safe management of patients with FVDs; this manual was subsequently adopted as the basis for WHO CMGs for frontline HCWs during the 2013–2016 EVD outbreak in West Africa. In addition, during the COVID-19 pandemic, the Ugandan MoH adapted, adopted and implemented existing WHO CMGs for management of COVID-19 patients.

Taking into consideration the potential challenges for CMG development and implementation in LMIC settings, this case study leveraged Uganda’s experience with CMGs from outbreaks of FVD and COVID-19 to explore the barriers and facilitators reported by healthcare practitioners during recent outbreaks. Specifically, this study assessed the contextual and supplementary factors that shape CMG development and implementation in the Ugandan context and asked when and why available CMGs are adopted or foregone by HCWs; how CMGs may be communicated to patients and the public to bolster confidence and trust; and how far guidelines accommodate different aspects and priorities of care.

## Methods

This qualitative in-depth case study examined the barriers and facilitators of CMG development and implementation within the Ugandan context. The study results are presented using the Consolidated Guidelines for Reporting Qualitative Research (COREQ).^27^ The study employed a qualitative deductive exploratory methodology^28^,^31^ well suited to explore cases where existing knowledge base is tested or revised. The data were analysed using a sequential thematic network analysis approach,^30 32^ which couples the exploration of the detailed ‘rich description’ of participants’ perspectives^33 34^ with network modelling to visualise the relationships between thematic codes. Unlike traditional thematic analyses, which primarily offers summarised description of data, thematic network analysis enables the synthesis of data through an exploration of the underlying structure of the codes. Like an exploratory factor analysis in statistics, thematic network analysis can identify underlying community structures (ie, cluster detection) within the thematic codes and, thus, group highly co-occurring codes together to create overarching themes. This method offers clarity in the research process and is replicable, allowing other researchers to follow and validate our work while maintaining the integrity of the qualitative data, boosting the reproducibility of our findings.

### Sampling and recruitment

Prior to enrolment, stakeholder mapping was conducted to categorise participants at different levels (ie, international, national and local). Sampling was conducted using purposive and snowball sampling techniques (see online supplemental file 1). Participants were eligible to participate if they were previously or currently involved in outbreak response or CMG development for FVDs and/or COVID-19. A total of 62 potential study participants from five general and nine regional referral hospitals, MoH headquarters and the WHO country office in Uganda were approached by both email and telephone and asked if they wanted to participate. Of these, 19 declined participation citing involvement in the 2022–23 SVD outbreak response at the time of data collection as justification. Participants who showed interest received consent forms via email or scheduled a face-face meeting for in-person provision of informed consent.

### Data collection

Forty-three interviews were conducted between August and December 2022 in English by a female and a male social scientist with MA degrees (OK and MA) and no prior relationship to the participants and no prior knowledge of the research topic, minimising bias. Participants had considerable knowledge and experience working in outbreaks either as CMG developers or front-line workers but had no prior knowledge about the researchers’ goal. Participants were interviewed online or in private rooms within their respective workplaces, with interviews lasting between 25 and 120 min; no other person was present during interviews. Unpiloted semistructured interview topic guides, developed collaboratively by the research teams based in Uganda and the UK, were used. The questions were carefully chosen in line with the study and informed by prior studies and systematic reviews conducted by the study team.^2^,^6^ Topics covered during interviews including subject matter knowledge, experiences with CMG development, perceptions on availability, access, adaptability and inclusivity of CMGs, and supplementary factors affecting implementation of CMGs (see onlinesupplemental files 2  3). With the onset of the SVD outbreak in September 2022, the topic guide was revised to include specific questions that captured information on how CMGs were applied in real time until data saturation was reached; no follow-up interviews were conducted. With permission, interviews were digitally recorded and transcribed verbatim, but transcripts were not returned to participants. Both face-to-face and virtual interviews were conducted privately after participants provided written consent; participants were compensated with 14 USD.

### Analysis

At the end of the first eight interviews, field notes were written and shared with a wider team to discuss initial ideas from the raw data and how potential biases, concerns, expectations and experience of the researchers could influence the analysis. On completion of interviews, transcripts were evaluated through thematic network analysis which involved: (1) data familiarisation from field notes and raw data; (2) deductive theme identification based on existing themes uncovered in the systematic evidence reviews of existing CMGs in LMICs^2^,^6^; (3) data coding of deductive themes; (4) inductive coding of in vivo codes arising from the descriptions of the real-world experiences of those working and managing HCID outbreaks; (5) organisation of codes and themes; and (6) exploration via network modelling. Themes and subthemes were compared with field notes to ensure credibility of the findings before final coding. Two researchers (SS and OK) coded the data using NVivo 12plus and NVivo 20, using a multi-theme coding method developed by SS.^30 32^ Codes were extracted from NVivo, transformed with co-occurrences between codes (ie, when a reference simultaneously was coded for two or more codes) calculated as edge weights (see online supplemental file 4). The data were imported into a social network programme (Gephi 0.9.5) to develop a visual representation of both the relationships between thematic codes and their importance in the overall network in line with prior network representation of qualitative data.^3133 35^,^37^ To reduce noise in the data and filter out all co-occurrences likely to have occurred by chance, the association rule ‘lift’ was used as a backboning filter (lift filter=1.0).^35 36 38^ Visual exploration (using the algorithm ForceAtlas 2) was used to analyse the interactions between codes, and modularity algorithms were used to determine which codes co-occurred more frequently with each other.^3237 39^,^42^ Additional graphs were developed using a Fruchterman Reingold algorithm^3739^,^41^ to visualise connections between individual codes and their closest neighbours. The graphs show the relationships of codes to each other, visualising the sum of references shared with one code as the size of the node (weighted degree), how often particular codes are discussed alongside each other in one reference (edge weights represented by thickness of links), the total number of references shared between all codes (centrality to the network), and which codes exhibit closer relationships with each other compared with the rest of the codes (modularity clusters). A Data Extraction Table, showing selected references, the applied codes, interpretation and community cluster, can be found in online supplemental file 5. While participants did not provide feedback on the findings, they were discussed with an expert panel prior to write up.

### Patient and public involvement

No patients were involved in this study. However, preliminary results were discussed with an expert panel prior to write up, and a knowledge exchange meeting was organised in Uganda where findings were discussed with stakeholders and participants. Results were also triangulated with results from two other case studies within the ESHCID project on barriers to implementation of CMGs for chikungunya in Indonesia and patient experiences of standardised care during FVD outbreaks in Uganda, Sierra Leone and Liberia.

## Findings

Forty-three participants, including 23 general medical personnel (ie, nurses and doctors (MP)), nine consultant physicians and surveillance officers from regional referral hospitals (CP), five MoH case management pillar members (CM), five members of MoH top management (TM) and one WHO country office official, participated in the interviews (see online supplemental files).

### Thematic clusters in the interviews

**Figure 1:**
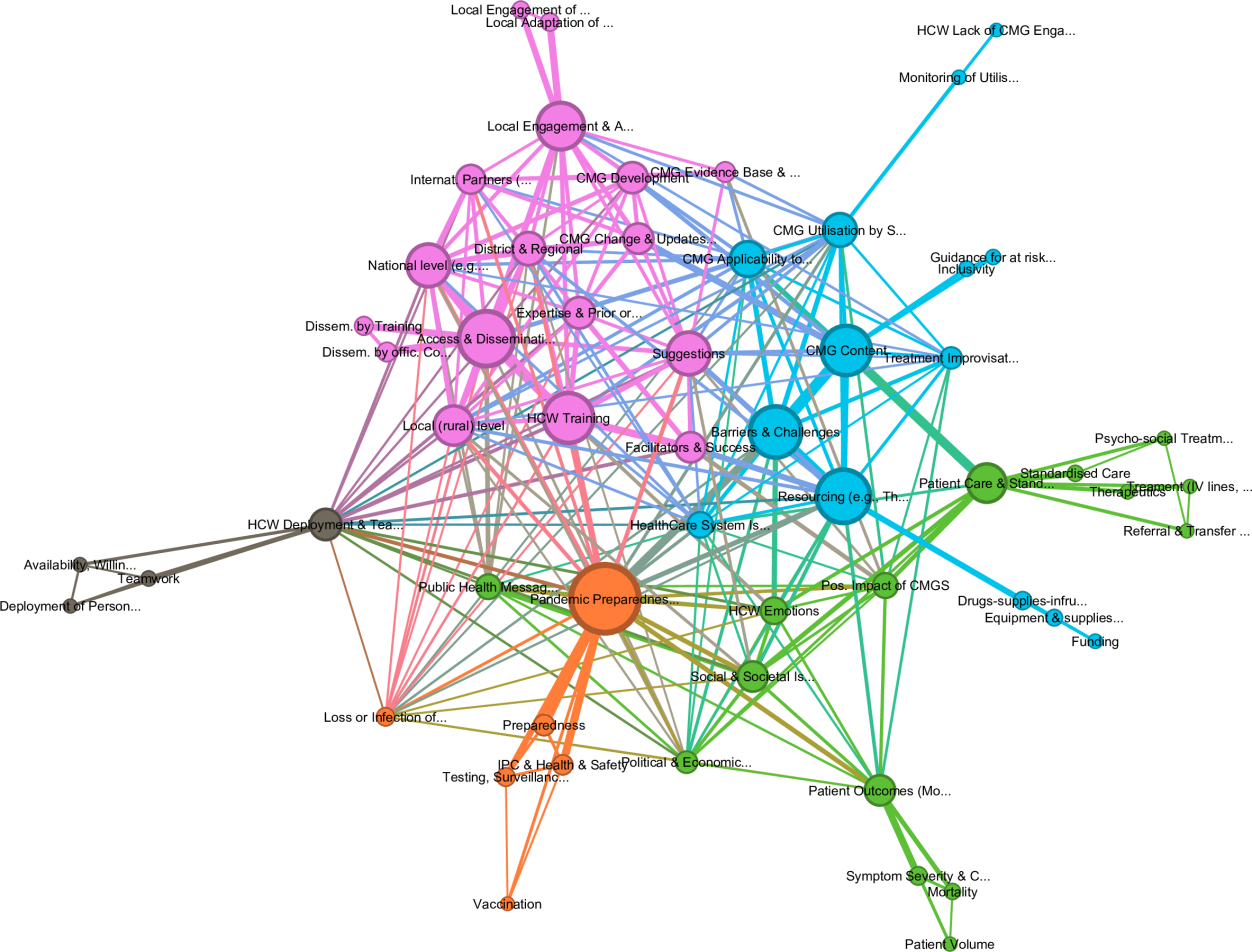
Graph of connections between all thematic codes, showing 58 codes, with five community clusters: *CMG development and dissemination* (violet, 30% of the codes in graph), *CMG applicability to patients, setting, and resources* (light blue,25%), *patient care outcomes and standardisation* (light green, 27%), *pandemic preparedness and response* (orange, 11%) and *workforce collaboration and engagement* (grey 7%). Lift, 1.0; graph density, 0.166; average weighted degree, 281.89; and modularity, 0.362 (showing moderate modularity). CMG, clinical management guidelines.

Fifty-eight thematic codes were identified and included in the initial thematic network graph (see figure 1). Two codes (COVID-19 and FVD) were excluded as their connections with other codes were heavily influenced by when the interview was conducted (see online supplemental file 7). This decision was taken as references discussing FVD were either most frequently coded against *pandemic preparedness and response*, at first related to a hypothetical future outbreak and after the start of the SVD outbreak in Mubende District in September 2022 related to the immediate outbreak response. On the other hand, references coded against COVID-19 were more equally spread across other codes. This strong co-occurrence between FVD with pandemic preparedness and response is visible in online supplemental file 7 which shows the most frequently associated codes with COVID-19 and FVD. After exclusion of these codes (see figure 1), modularity calculation for cluster detection showed moderate modularity (Leiden algorithm, 0.362), resulting in five thematic topic clusters in the thematic codes: *CMG development and dissemination* (violet, 30% of the codes in graph), *CMG applicability to patients, setting, and resources* (light blue,25%), *patient care outcomes and standardisation* (light green, 27%), *pandemic preparedness and response* (orange, 11%) and *workforce collaboration and engagement* (grey 7%). The following sections will discuss the five thematic clusters in turn before highlighting the most frequently discussed barriers and facilitators for the implementation of CMGs in Uganda.

#### clinical management guideline (CMG) development and dissemination

The most important themes in this cluster consisted of *access and dissemination of guidelines*, *training of HCWs*, and *local engagement and adaptation* in healthcare facilities, followed by the need for *CMG development and updates* that reflect the *existing evidence base and research*. Figure 1 illustrates the close connection of these codes alongside their importance to the network. Relatedly, the cluster also discussed the importance of *updating and adapting CMGs* by engaging with and meeting the needs of those with *expertise and professional experience from prior outbreaks* who are required to implement the guidelines, thus ensuring the inclusion of stakeholders at the *local, district and regional levels*, alongside *national (MoH*) and *international partners (CDC, WHO, NGOs*). The presence of the code *facilitators and success* in this cluster indicates that many codes in this cluster were also discussing facilitators for the successful implementation of CMGs. The codes in this cluster are visible in online supplemental file 6 sorted by their relative importance to the network (by weighted degree).

Many participants expressed that, while guidelines developed for high-income countries were challenging to adapt to resource-constrained healthcare facilities, Ugandan CMGs were developed with minimal input from local stakeholders able to adapt guidelines to available local resources. Consequently, many participants felt that too much emphasis was placed on international research in CMG development, with insufficient guidance on how to incorporate local evidence, particularly from personnel who might encounter outbreaks and changing case presentations first. In addition, participants routinely emphasised the importance of CMGs being guided by existing evidence and research collected in Uganda, which would enable the rapid integration of new knowledge regarding symptomatology or treatments into existing guidance and lead to increased use of CMGs by staff (see figure 1). For example, one participant noted that due to guidance overemphasising bleeding as a primary sign in viral haemorrhagic, early detection of cases during the West Africa EVD outbreak was likely ineffective given a small percentage presented with this sign.

Participants also frequently reported that the availability of CMGs was inconsistent, with more frequently occurring or internationally prioritised HCIDs (eg, Ebola disease and COVID-19) being prioritised over less common HCIDs (eg, Marburg disease). Logistical factors (eg, scarcity of print copies, limited internet access and limited training for HCWs) were highlighted as barriers to CMG implementation. Many described that HCWs were often unaware of guidelines, facing challenges in accessing them due to slow and inconsistent dissemination and limited announcements. For example, rural healthcare facilities lacked printed copies and frequently could not access online resources, which reportedly hindered effective communication across healthcare facilities, access to health records, dissemination of guidelines and attendance at virtual seminars. Such limitations in availability of CMGs for FVDs disproportionately affected lower-level rural facilities and, as highlighted by one participant, were especially detrimental to pandemic response since outbreaks tended to first occur in rural areas of affected countries.

Alongside dissemination challenges and lack of internet access, inadequate applied training to disseminate guidance was discussed as a central issue. The absence of applied training on guidelines and dedicated platforms for accessing them was also associated with colleagues seeking false information and subsequently applying questionable treatments for COVID-19 with repurposed therapeutics (eg, ivermectin, hydroxychloroquine) even after warnings from WHO, CDC and Ugandan MoH guidelines. One participant suggested that adherence to guidance (eg, failure to manage electrolyte balance) was less often due to lack of knowledge and rather due to a lack of practical training and continued mentorship at the healthcare facility. Also, many participants mentioned that colleagues were unlikely to keep abreast of the latest evidence during their personal time, which exacerbated the issue, further highlighting the importance of training.

#### Clinical management guideline (CMG) applicability to patients, settings and resources

**Figure 2:**
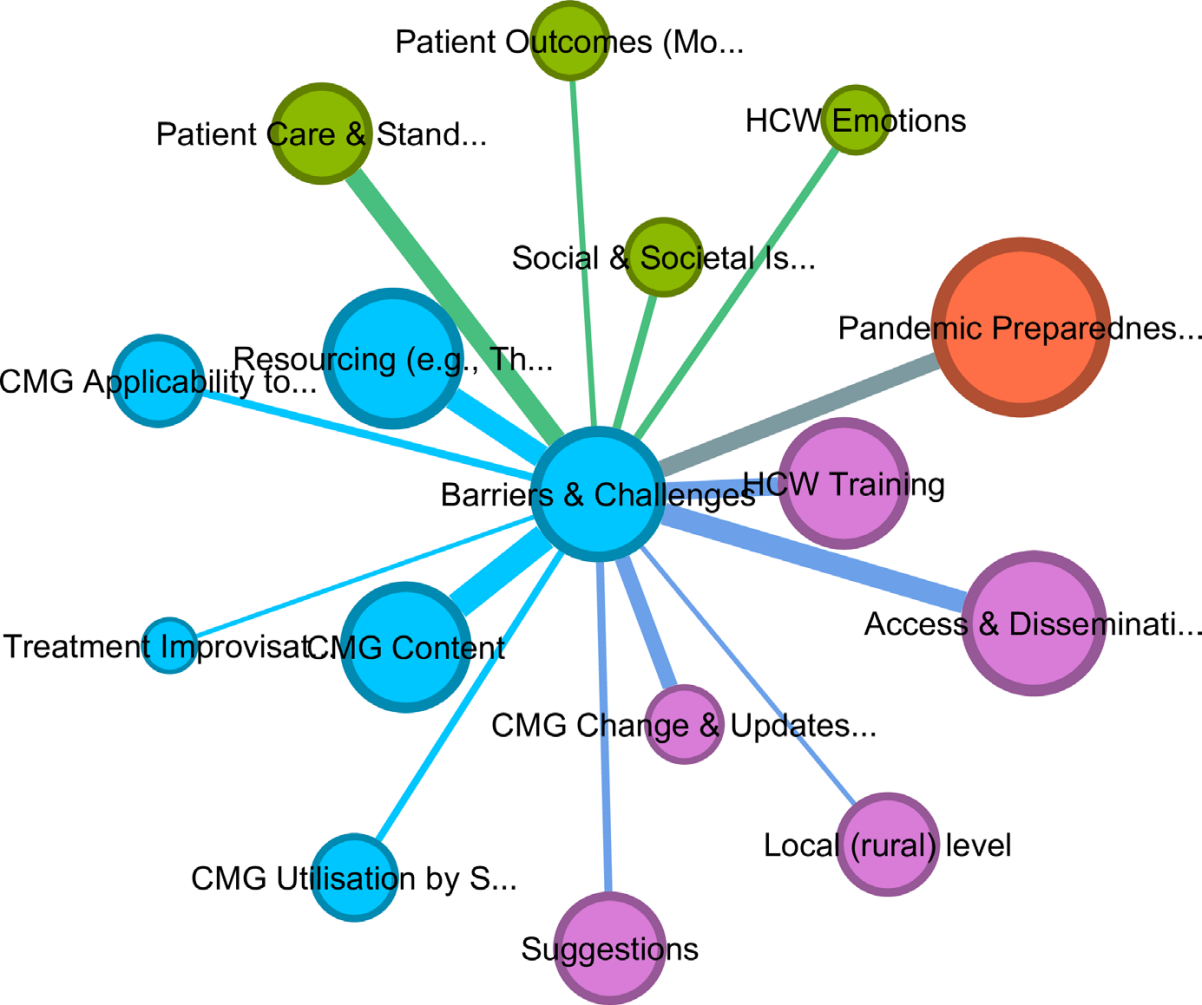
Overview of codes sharing most references with barriers and challenges to the implementation of CMGs (adjacency and thickness of links) using a Fruchterman Reingold layout. Lift, 1.0; edge weight, 40; graph density, 0.125; average weighted degree, 166.75. CMG, clinical management guideline; HCW, healthcare worker.

The thematic cluster *CMG applicability to patients, setting and resources* consisted of 14 codes that were more interconnected than other codes in the network (see online supplemental file 6). Codes in this cluster predominantly addressed the need to adapt CMG content to particular patient groups and local settings in line with available resources (eg, therapeutics, equipment, staff) and highlighted the impact of healthcare system issues. The code *barriers and challenges to CMG implementation* also occurred in this cluster, suggesting that most references mentioning barriers also referenced these codes (see figure 2 for an isolated graph of barriers).

Resourcing issues and CMG utilisation challenges (due to either the lack of suitable guidance, HCWs being unaware of them or HCWs choosing to ignore them) were often discussed alongside issues within the Ugandan healthcare system. Participants frequently recognised a discrepancy between nationally adopted guidelines and the logistical and resourcing limitations on the ground, which hinder universal CMG implementation. Due to content that did not take into consideration available resources in local settings, CMG utilisation by staff was inconsistent and reportedly led to treatment improvisation. Participants also discussed frequently that CMGs did not equally address all patient groups. COVID-19 guidelines—owing to a substantial amount of evidence-based guidance from international bodies—were perceived as inclusive of different at-risk groups (eg, HIV/immunocompromised individuals, pregnant women, children, elderly) and were frequently updated to reflect advancements in pathogen and therapeutic evidence. In contrast, CMGs for FVDs were reported as needing greater inclusivity to adequately cover at-risk patient groups. As well, many highlighted the urban-rural divide, noting that severe cases are transferred to regional hospitals equipped to monitor oxygen, fluid and electrolyte balance, dialysis, etc, while lower-level rural facilities often lacked access to these resources and faced challenges with accessing transportation to transfer patients. Universal CMG implementation was reportedly further inhibited by differences between private and public healthcare facilities, where private facilities might distribute therapeutics more quickly or implement different infection prevention and control (IPC) measures, such as reusing single-use personal protective equipment (PPE) or using non-recommended medications.

#### Patient care outcomes and standardisation

Themes in this cluster centred around *Patient Care & Standardisation*, *Patient Outcomes*, and *social, societal and political issues (see[Supplementary-material SP6]* online supplemental file 6). Interestingly, many of these codes were also connected with the *positive impact of CMGs* and *HCW emotions* highlighting how adherence to CMGs and resulting adequate standardised patient care would lead to positive effects on patients and staff but also on the public and society. It is important to note, however, that this cluster shows a wide distribution of codes across the network (figure 1), highlighting the interconnectedness of individual themes with other clusters across the graph. For example, *patient care & standardisation* is strongly linked to *CMG content and resourcing*, while *public health messaging* and *societal, political and healthcare system issues* are strongly related to *pandemic preparedness and response; subcodes include patient outcomes* and *patient care & standardisation*.

CMGs were routinely regarded as important for increasing standardisation of care, for promoting shared understanding between HCWs and to reducing negative patient outcomes. Moreover, adherence to guidelines and standards of care was associated with reduced fear and anxiety of HCWs who were working in HCID outbreaks. Some participants also highlighted indirect positive effects of adherence to CMGs, including reducing stigma and mistrust in the population and improving the population’s adherence to public health measures, such as coming forward for testing.

Social, societal, political and economic realities on the ground were often discussed as limiting effective outbreak response for COVID-19 and FVD and impairing effective patient care. For example, lack of resources exacerbated by existing healthcare disparities across the country led to disruption of essential services for other diseases (eg, HIV, malaria) and downstream adverse patient outcomes. Participants, therefore, frequently highlighted the need to be able to continue other essential healthcare services during outbreaks.

An impaired ability to provide standardised care was also routinely linked to social and political issues that were only tangentially related to the healthcare system. Many identified the lack of adherence to guidelines among the population, which hindered the effective treatment of patients. For example, participants routinely described that a lack of outbreak preparedness, a reluctance to engage with scientific information and generally low medical literacy in the population (and even in some healthcare staff) impeded their ability to implement guidelines and carry out their job. This issue was reportedly exacerbated by widespread stigma in the population, which perpetuated misperceptions, myths and conspiracy theories (eg, ‘witchcraft’, ‘government harvesting organs’ or ‘killing people to access natural resources’), making the work of HCWs difficult and, at times, dangerous. A few participants highlighted that this adverse environment directly impaired their ability to adhere to CMGs, such as recommended burning of recovered patients’ possessions (often without compensation to patients for the loss of their possessions) aimed to minimise risk of HCID transmission from treatment units when patients are discharged back to the community. Additional problems undermining effective implementation of CMGs highlighted by some participants included delayed payment of HCWs, bureaucratic politics between different healthcare pillars, resource wastage (eg, PPE, medicines) and even occasional corruption.

Correspondingly, *public health messaging* was a recurring theme in this cluster, as inadequate public health adherence by the population reportedly impaired patient care and thus led to adverse patient outcomes. Considering these issues, participants described the need for public health communication templates to be disseminated alongside CMGs to enable HCWs to integrate understanding by community leaders, patients’ relatives and the population at-large in their approach to management of patients. Figure 1 highlights that many of the references in this code also discussed *pandemic preparedness and response* and *rural (local) level*, underscoring the importance of such issues in rural regions of Uganda.

#### Pandemic preparedness and response

Codes in the thematic cluster of *pandemic preparedness & response* included *preparedness* for HCID outbreaks; issues of *IPC & health and safety; loss or infection of HCWs; testing, surveillance & early warning*; and *vaccination* (online supplemental file 6). While many of these codes appear unrelated to CMG implementation, participants frequently discussed overall pandemic preparedness and response in the context of their challenges in adhering to or implementing CMGs.

Logistical issues around surveillance and testing and shortage of staff resulting in breaches of IPC measures were commonly cited as barriers to effective guideline implementation, for example, lack of staff resulted in longer duration in treatment units than advised. While COVID-19 surveillance infrastructure was described as fairly robust, the limited availability of national laboratories equipped to process samples from patients with FVD reportedly led to frequent diagnostic delays, which impeded guideline adherence and increased infection risk among staff. This situation was exacerbated by resource shortages; for instance, staffing shortages required HCWs to remain on wards longer than recommended, which impacted both supportive care provision and IPC protocol adherence.

As most FVD outbreaks were seen as predominantly occurring in rural areas and often close to borders, some participants suggested using mobile lab equipment to expedite testing and community surveillance. They also recommended training of a cadre of readily deployable response personnel to support and train local health facility staff. Given the rural settings of many HCID outbreaks and potential for cross-border spread, some participants highlighted the need for CMGs in outbreak response to consider transmission dynamics across borders. In addition, they emphasised the importance of early capacity building and ongoing clinical education in HCID clinical management to mitigate skill loss and prevent staff turnover. Lastly, participants routinely discussed the need for public health messaging being developed and updated alongside CMGs to ensure public adherence to guidelines.

#### Workforce collaboration and engagement

The last cluster, *workforce collaboration and engagement,* comprised mostly of inductive codes that arose from the interviewees responses, as they routinely discussed issues around *interprofessional collaboration* between HCWs from different pillars, the *deployment of personnel* to HCID treatment areas, and the *availability or willingness of HCWs* to work on HCID wards (online supplemental file 6).

Several participants, particularly nurses, mental health providers and those in more rural areas, discussed how lack of dissemination of CMGs to personnel from different pillars (eg, case management; mental health) and in different levels of the healthcare system would result in ineffective inter-professional collaboration and effective working among pandemic and outbreak responders. Simultaneously, a general lack of knowledge about the likelihood of acquiring infection, especially in rural areas, led to an unwillingness of HCWs to work in HCID treatment units. This perspective was rooted in both the lack of knowledge, inexperience or lack of preparedness of HCWs—especially if they had not received training—as well as the lack of knowledge in rural communities, which created dangerous situations for HCWs (eg, threats of violence) and further decreased the willingness of HCWs to work in treatment units.

Reduced availability of HCWs willing to work in treatment units increased pressures on those HCWs who were working in HCID treatment units, which reportedly corresponded to burn out and risk for breaches in CMG adherence. To counteract this effect, the dissemination and training of CMGs at every level of healthcare providers from Village Health Teams to clinicians were described as paramount to the implementation of CMGs and the increased willingness of HCWs to work at the frontline. Adequate public health messaging was frequently suggested as enabling HCWs to do their job. The closeness of these themes to each other is visible in figure 1, which shows how closely connected the themes of this cluster are to *public health messaging*, the *rural level* and *loss or infection of HCWs*.

### Barriers to the implementation of clinical management guidelines (CMGs)

Figure 2 depicts the codes most frequently discussed across all clusters alongside barriers and challenges in CMG implementation; the relative importance of these codes, represented by their weighted degree, is displayed in online supplemental file 9. The graph highlights that *CMG Content*, *HCW Training*, *Resource Limitations*, and *Social and Societal Issues* are most closely connected to these barriers. Specifically, CMG content that did not reflect the needs of at-risk patient groups, the Ugandan healthcare system or rural settings with limited resources was frequently cited as a reason why HCWs improvised treatments or did not use guidelines. Similarly, the lack of practical, ongoing training for HCWs, along with infrequent updates and patchy dissemination—especially in rural healthcare facilities where CMGs were often unavailable—was also highlighted as critical barriers.

The graph further underscores the connection between *Social and Societal Issues* (eg, community non-compliance with guidelines and attacks on HCWs) and the difficulty in providing standardised patient care, which negatively impacted patient outcomes and influenced HCW emotions (eg, fear and anxiety about infection or community hostility). It is notable that many of these issues were linked to the rural areas where HCID outbreaks were reported to occur more frequently and where preparedness was lowest. Lastly, many participants highlighted the overall limited outbreak response preparedness—specifically inadequate IPC measures, surveillance and testing for HCIDs other than COVID-19—as a significant barrier to CMG implementation in Uganda.

### Suggestions and facilitators for the implementation of clinical management guidelines (CMGs) for high consequence infectious disease (HCIDs)

**Figure 3:**
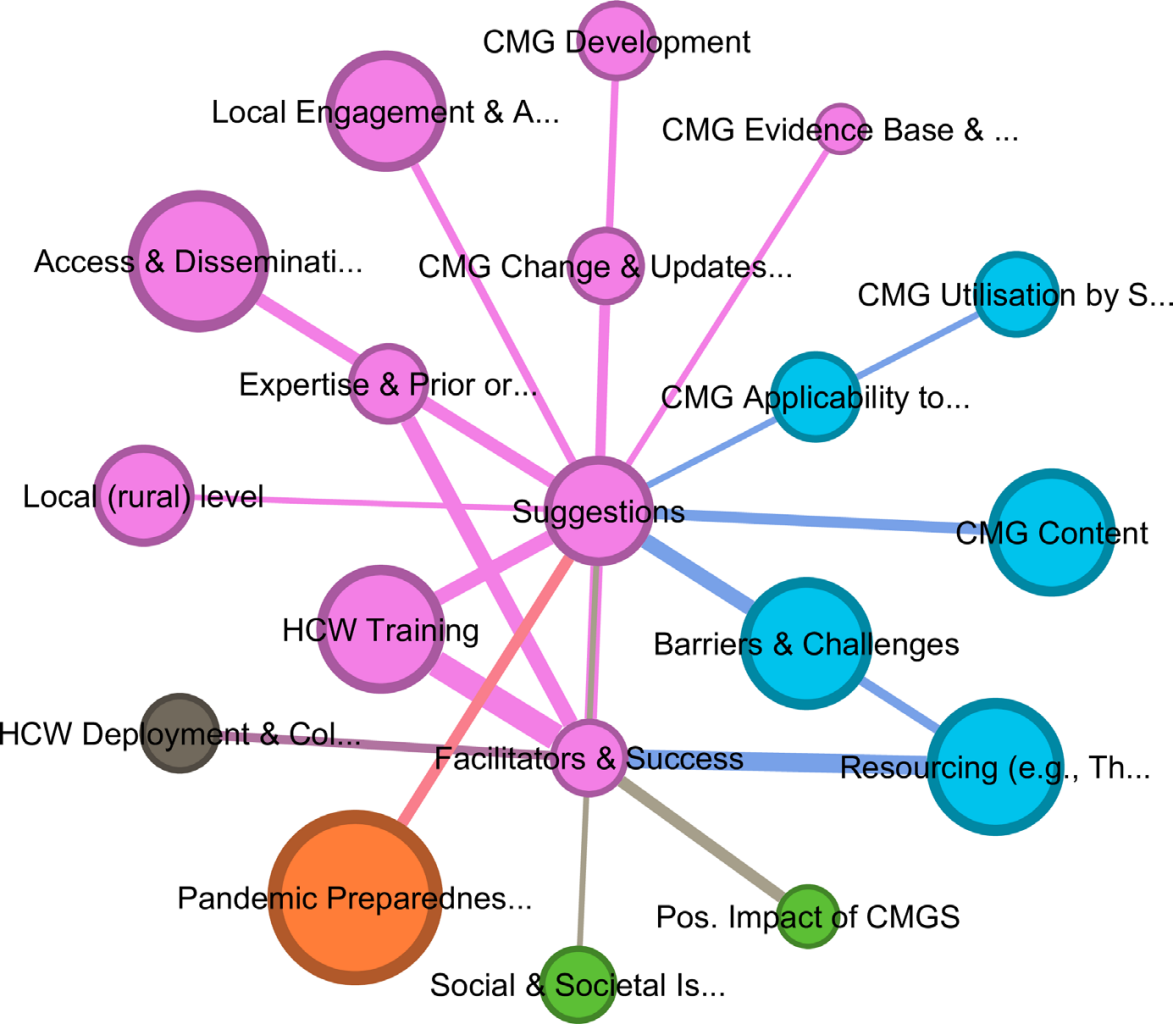
Thematic codes most frequently associated with suggestions and facilitators for the implementation of CMGs using a Fruchterman Reingold layout. Lift, 1.0; edge weight, 15; graph density, 0.123; average weighted degree, 91.36. CMGs, clinical management guidelines; HCW, healthcare worker.

Figure 3 illustrates the codes most frequently connected to *Suggestions* and *Facilitators* for enhancing CMG implementation. As shown in online supplemental file 10, the high weighted degree of these codes reveals that HCW training in CMGs, adequate *resourcing of therapeutics, equipment, and staff*, and the involvement of personnel with prior HCID response experience in CMG development were the codes most strongly linked to both suggestions and facilitators. To ensure harmonisation of training, standardisation of care across the healthcare system and increased educational opportunities for staff in rural facilities with fewer resources, most participants emphasised the benefits of hands-on cascade training with on-site local personnel responsible for training and championing CMG adherence. Training, in particular, was reported to increase confidence and self-efficacy, which was related to more rigorous implementation of CMGs. Rehearsals and simulations conducted by experts experienced in HCID management were frequently associated with successful CMG implementation and compliance.

Nearly all participants suggested improvements for better access to and dissemination of CMGs to support HCW uptake and utilisation. Recommendations included: (a) enhanced internet access alongside ‘living guidelines’ to allow virtual, real-time access to guidelines, integrated patient health records and communication; (b) dissemination of print materials such as pamphlets, posters and training guides within facilities; and (c) hands-on practical training on guidelines for HCWs. Participants noted that internet access alone would not ensure CMG implementation; instead, dedicated online portals, email communications and social media campaigns would be necessary for rapid, pervasive dissemination of updates.

Recognising the challenges of providing adequate healthcare resources in LMICs, participants recommended that guidelines be adapted to reflect local resource conditions while aligning the procurement of therapeutics, equipment and surveillance with suggested guidelines. The involvement of local stakeholders in CMG development and related research was frequently discussed as essential for improving guideline applicability to local contexts, enabling rapid responses to outbreaks and including at-risk populations (eg, children, elderly, immunocompromised, pregnant women). While they highlighted the need for collaboration among international partners, medical-regulatory bodies, the MoH and local stakeholders to ensure timely updates with the latest evidence on pathogens and treatments, participants also stressed the importance of local stakeholders and local research efforts into HCIDs to increase HCW engagement and ensure guideline relevance to the LMIC context. Some discussed the need to integrate Ugandan research sites into clinical trials to increase buy-in and adaptability to context. Figure 3 clearly emphasises the importance of adapting CMGs and training delivery to the local, rural levels of the Ugandan healthcare system. One doctor working in a remote district during COVID-19 summarised the range of suggestions for enhancing CMGs in Uganda:

*First, all the stakeholders who develop guidelines should resist from copy and paste […], so, we read the guidelines [and] tailor them to what works for us. Second, [involve] the people who are managing patients on the ground, […] no matter the qualification. Even if someone is an enrolled nurse involved in the day-to-day management of patients, they practically know more than the professor who will pass there once in two months. Third, when these guidelines are passed on, it should be for practical purposes not for accountability purposes*. (MP13)

Lastly, participants frequently discussed other aspects related to successful CMG implementation, which extended beyond clinical management to pandemic preparedness and response. These additional topics included the integration of public health messaging and initiatives to dispel myths and misconceptions in local communities, as these factors affect both HCW ability to perform their duties and their willingness to work on HCID units and adhere to guidelines during outbreaks. Box 1 provides an overview of concrete recommendations and suggestions from this research.

Box 1 **Key Recommendations for clinical management guideline (CMG) implementation in Uganda**

**Recommendations for CMG updates, dissemination and monitoring**

Include practitioners and local stakeholders in CMG development to ensure representation of views of personnel from resource-constrained settings, particularly rural settings from where many high consequence infectious diseases (HCIDs) may initially emerge.

Identify and address discrepancies between recommendations and available resources to ensure availability of resources for quality care of patients during HCIDs.

Continuously review and update Filovirus disease CMGs to include new evidence and management options that emerge from clinical trials.

Increase access to guidelines through provision of living guidance, hard copies, posters, webinars and local facility CMG champions.

Integrate local research sites into HCID trials and incorporate local stakeholders to increase buy-in and adaptability to context.

Develop and invest in public health messaging alongside CMGs to address stigma in the populace, aversion to accessing care during HCID outbreaks and willingness of healthcare workers (HCWs) to deploy to work in HCID treatment units.

**Recommendation for HCW training of HCID CMGs**

Deliver practical training on HCID CMGs to HCWs throughout their educational and professional development with periodic follow-up to counteract reduced willingness of HCWs to work in epidemic/pandemic response.

Use mentorship and cascade training (delivered by experienced personnel) to model clinical care, monitor compliance and increase self-efficacy and HCW preparedness.

Provide training on CMGs that is hands-on, practical and skills-based, using rehearsals and simulations.

Monitor and evaluate CMG utilisation and delivery of standards of care.

**Recommendations for resourcing of supportive care and research**

Align procurement of therapeutics and equipment and staff allocation with the guidelines by integrating them into the guideline development process.

Create readily deployable response teams to bolster and support local health facility staff and involve more physicians in outbreak response.

Invest in mobile lab equipment and personnel to deploy to lower-level facilities aimed at facilitating faster testing and community surveillance.

Improve communication and coordination between different partners involved in outbreak response, including continuous updates of medical staff on new information and trends through virtual and non-virtual channels.

## Discussion

The analysis revealed several key themes related to the implementation of CMGs in Uganda. Five thematic clusters were identified: *CMG development and dissemination; CMG applicability to patients, setting and resources; patient care outcomes and standardisation; pandemic preparedness and response*; and *workforce collaboration and engagement*.

The participants highlight the importance of CMGs as the first—and often only—defence against an HCID outbreak, providing clinicians with the necessary standardised guidance to administering supportive care for HCID patients,^17^ particularly in rural healthcare settings where HCID outbreaks may be more common, but healthcare facilities are least prepared. However, the study also clearly illustrated some of the key conceptual, logistical and practical barriers inhibiting the implementation of CMGs in LMIC outbreak contexts. While previous research during the 2013–2016 EVD outbreak in West Africa demonstrated the importance of optimised supportive care—showing higher case fatality rates among patients treated in resource-constrained settings with limited access to technologies^43 44^—the presented findings highlight the complexity of barriers to implementation of CMGs for standardised care in LMIC contexts. Specifically, by asking healthcare practitioners about the barriers and facilitators to their utilisation and implementation of CMGs, the study was able to go beyond the proximal causes attributed to issues with the availability, inclusivity, quality and applicability of CMGs for HCIDs.^2^,^6^ Expanding on evidence that CMGs are hindered by limited awareness of existing guidelines by HCWs,^45^ limited access to treatments and equipment,^12^ the results show that many of these problems are tightly connected to logistical, social or practical issues. For example, inconsistent CMG implementation or outright improvisation of treatments was not only linked to a lack of applicability of CMGs to available resources or particular at-risk patient groups, but routinely tied to the difficulties adapting CMGs to the layered healthcare system with differing access to resources in rural/urban but also private/public settings. As such the findings suggest that addressing resource constraints alone may not result in successful implementation if existing healthcare disparities are not considered to ensure the standardised provision of care across the country. Similarly, while increasing access to CMGs through better dissemination would be beneficial, the analysis demonstrates that such endeavours are likely insufficient if HCWs are not simultaneously provided with hands-on training on the guidelines. Increasing internet access was discussed as important to access CMGs in rural areas, but increased access to social media could further exacerbate many of the prevailing myths and misperceptions in the population, resulting in more violence and stigma towards HCWs and unwillingness of HCWs to work on HCID treatment units.

The results therefore emphasise the complex relationship between some of the proximate causes hindering CMG implementation (eg, lack of access, insufficient training and slow updating) and more ancillary factors that exacerbate many of the underlying difficulties for pandemic response and preparedness in LMIC contexts. CMGs therefore are an important tool to improve equity in access to evidence-based care in outbreaks,^18 19^ but HCWs will be unable to implement the standards of care necessary to quell HCID outbreaks if these peripheral underlying limitations to availability, accessibility and applicability are not addressed.

Nevertheless, the findings also highlight a range of facilitators for the successful adoption and implementation of CMGs by HCWs in Uganda. Specifically, improvements in applied training for HCWs in CMGs alongside to efforts to monitor implementation and the inclusion of local stakeholders in CMG development and modification to provide contextual relevance to at-risk patients, local settings and resource constraints, as well as advances in access and dissemination of CMGs, were the codes most strongly linked to facilitators. In line with suggestions for guidelines to be contextually relevant, accessible for use by front-line clinicians and inclusive of vulnerable patient groups,^18 19^ the findings underline the importance of adapting international guidelines to local settings. However, alignment of guidelines to the locally resources requires careful identification of the concrete discrepancies between recommendations and the available resources is required to target help ensure availability of needed resources for the provision of quality care of patients during HCIDs.

### Limitations

This study used a convenience sample of personnel with considerable working experience in COVID-19 and/or FVD outbreaks in Uganda. While it is, therefore, not generalisable to a global context, our study collated a range of concrete and actionable recommendations from the interviews, which may be illustrative for those seeking to develop practical CMGs for supportive care in HCID outbreaks in other settings. Furthermore, the number of interviewees was reduced due to deployment of several potential participants to the SVD outbreak in Uganda, starting in September 2022. Nevertheless, the serendipitous inclusion of various participants, who were able to be interviewed despite being deployed during the outbreak in Uganda, enabled data collection as an HCID outbreak was underway. The methodology used for our analysis should also be considered a limitation of the study; while useful for analysis of larger qualitative datasets, it is important to interpret the networks as illustrating relationships without suggesting causality. As the links between codes are undirected, based on code co-occurrence, they can only show which themes frequently co-occurred together and require the thematic analysis on which they are based to provide clarification. Nevertheless, the method effectively illuminates thematic links and clusters that might remain obscured with conventional thematic analysis techniques.

### Key recommendations

The presented findings from the Ugandan experience of the COVID-19 pandemic and the SVD outbreak in 2022 inform a range of general recommendations for the successful implementation of CMGs for HCIDs (see Box 1). By focusing greater attention on the realities of guideline adoption and implementation, insights from this work can therefore not only inform efforts to improve implementation of supportive care guidelines in future HCID outbreak scenarios, but also provide recommendations for supranational, governmental and non-governmental organisations who often advise and fund CMG development in LMICs.

## Conclusion

This in-depth case study of Uganda aimed to explore the barriers and facilitators for successful implementation of CMGs for HCIDs in Uganda. It highlights the significant obstacles to implementing CMGs in LMIC contexts, where outdated guidelines, poor dissemination and training deficits exacerbate underlying healthcare disparities and resource constraints. However, the research also demonstrated the need for CMG development in LMICs to extend beyond adaptation of international clinical evidence but requires adjusting for local epidemic and pandemic preparedness and response capabilities and demands. Effective CMG implementation, therefore, necessitates involvement of local stakeholders to help contextualise guidelines and use of both virtual and print means, supported by hands-on cascade training, for the wide dissemination of living guidelines for HCID management to front-line health workers. Besides highlighting barriers to CMG implementation, the research also offers actionable recommendations to enhance CMG implementation which underscore the necessity of integrating local stakeholders to ensure guidelines are reflective of the reality of the local health system, applicable and inclusive of resource-constrained settings, disseminated widely and in a living manner and complemented by hands-on cascade training. While these findings from Uganda are not universally applicable, they offer valuable insights for LMICs to improve HCID outbreak responses and for organisations involved in guideline development and funding.

## Supplementary material

10.1136/bmjph-2024-001165 online supplemental file 1

10.1136/bmjph-2024-001165 online supplemental file 2

10.1136/bmjph-2024-001165 online supplemental file 3

10.1136/bmjph-2024-001165 online supplemental file 4

10.1136/bmjph-2024-001165 online supplemental file 5

10.1136/bmjph-2024-001165 online supplemental file 6

10.1136/bmjph-2024-001165 online supplemental file 7

10.1136/bmjph-2024-001165 online supplemental file 8

10.1136/bmjph-2024-001165 online supplemental file 9

10.1136/bmjph-2024-001165 online supplemental file 10

## Data Availability

All data relevant to the study are included in the article or uploaded as supplementary information.
