# Supplementary material for: ‘Involve those who are managing these outbreaks’: stakeholders’ perspectives on the barriers and facilitators to the implementation of clinical management guidelines for high-consequence infectious diseases in Uganda—a thematic network analysis
Source: BMJ Public Health. 2025 Feb 13;3(1):e001165. doi: 10.1136/bmjph-2024-001165 (PMC11843484; doi:10.1136/bmjph-2024-001165)
Supplement: online supplemental file 1 [file bmjph-3-1-s001.pdf]

Level	Institutional level	Category	Participants	Gender (m/f)	Age Range	Educational Level	Years of work experience	High Consequence Infectious Disease Experience
**International**	WHO	International (Int)	1	(1/0)	40-50	Masters	10+	ECM
**National**	Ministry of Health	Top Management (TM)	4	(4/0)	50-75	Masters	10+	ECM
**National**	Ministry of Health	Case Management (CM)	6	(3/3)	34-56	Masters (4)<br>PhD (2)	Oct-27	ECM
**Local**	Regional Referral Hospital	Consultant Physicians/surveillance officers (CP)	9	(8/1)	35-50	Masters (7)<br>Diploma (2)	Jul-24	ECM (3)<br>CM (1)<br>CE (2)<br>C (2)
**Local**	Hospital	Medical Personnel, e.g., Nurses, Doctors (MP)	23	(16/6)	28-61	Diploma (10)<br>Bachelors (7)<br>Masters (6)	Mar-35	ECM (2)<br>CM (3)<br>C (6)<br>E (2)

Supplementary Table providing an Overview of interviewed participants, including Abbreviations: **C-COVID-19: E-Ebola: M-Marburg**
