# Supplementary material for: ‘Involve those who are managing these outbreaks’: stakeholders’ perspectives on the barriers and facilitators to the implementation of clinical management guidelines for high-consequence infectious diseases in Uganda—a thematic network analysis
Source: BMJ Public Health. 2025 Feb 13;3(1):e001165. doi: 10.1136/bmjph-2024-001165 (PMC11843484; doi:10.1136/bmjph-2024-001165)
Supplement: online supplemental file 4 [file bmjph-3-1-s004.pdf]

ID	Label	Original Title		1 : Access & D	2 : Dissem. by	3 : Dissem. by	4 : Public Hea	5 : Barriers &	7 : CMG Appli	8 : CMG Char	9 : CMG Cont	10 : Guidanc	11 : Inclusivit	12 : CMG Dev	13 : CMG Evic	14 : CMG Util	15 : HCW Lac	16 : Monitorir	17 : Local Eng	18 : Local Ada	19 : Local Eng	20 : Pos. Impa	22 : COVID-1	23 : FHVs	25 : Expertise	26 : HCW De	27 : Availabili	28 : Deploym	29 : IP Collab	30 : HCW Em	31 : HCW Tra	32 : Loss or In	35 : District &	36 : Internat.	37 : Local (rur	38 : National	39 : Facilitato	40 : HealthCa	41 : Pandemi	42 : IPC & He	43 : Prepared	44 : Testing, S	45 : Vaccinat	46 : Patient C	47 : Psycho-s	48 : Referral &	49 : Standard	50 : Therapeu	51 : Treament	52 : Patient O	53 : Mortality	54 : Patient V	55 : Symptom	56 : Political &	57 : Resourci	58 : Drugs-su	59 : Equipmen	60 : Funding	61 : Social & S	62 : Suggestic	63 : Treatmen
			1245	396	115	133	83	511	110	254	378	35	43	116	162	97	21	36	153	95	78	107	209	262	196	82	25	21	51	110	319	25	104	106	125	171	381	57	276	110	132	80	21	360	28	52	71	41	25	135	51	22	91	41	234	73	46	28	169	135	64
1	Access & Dissemination o	1 : Access & D	396	396	108	93	51	139	39	69	81	5	3	30	20	45	16	13	78	66	18	33	111	99	39	24	10	8	9	19	138	15	46	37	59	76	100	15	86	31	42	23	10	70	8	14	26	9	2	16	7	1	9	14	38	14	4	4	37	48	18
2	Dissem. by offic. Comms	2 : Dissem. by	115	108	115	31	11	37	8	20	27	1	1	11	5	15	10	3	17	16	1	4	28	22	13	4	2	2	1	2	55	2	12	17	16	26	25	4	16	2	10	3	3	11	1	0	1	2	0	0	0	0	0	2	8	3	2	1	5	11	5
3	Dissem. by Training	3 : Dissem. by	133	93	31	133	4	40	5	21	31	2	1	6	4	17	4	8	40	35	8	5	28	35	29	7	4	2	2	6	109	3	26	12	30	29	40	6	26	9	15	4	3	19	1	6	5	2	3	6	2	1	3	1	22	8	5	7	7	19	2
4	Public Health Messaging &	4 : Public Hea	83	51	11	4	83	31	6	16	13	2	1	4	0	3	1	0	14	9	7	9	24	35	10	6	4	1	2	12	13	2	7	4	12	16	21	5	38	13	18	20	1	15	3	1	3	1	0	10	4	1	7	15	11	1	1	1	51	7	0
5	Barriers & Challenges	5 : Barriers &	511	139	37	40	31	511	59	102	168	13	22	30	46	55	16	17	43	25	24	27	125	85	53	29	15	8	15	61	115	11	37	26	42	52	72	39	113	46	53	25	13	127	10	27	19	17	18	47	17	9	28	25	145	46	31	17	62	59	40
6	CMG Applicability to Setti	7 : CMG Appli	110	39	8	5	6	59	110	19	60	6	5	26	20	21	3	3	28	17	17	7	65	33	7	5	2	1	3	5	23	3	17	16	28	27	21	12	18	12	3	4	0	33	2	8	6	2	1	7	4	0	3	4	31	7	6	3	14	16	16
7	CMG Change & Updates o	8 : CMG Char	254	69	20	21	16	102	19	254	85	10	11	42	32	14	4	7	55	27	36	11	48	49	41	10	3	2	7	12	35	5	24	36	21	35	55	9	53	21	17	8	11	52	4	1	17	15	2	19	6	1	13	7	36	7	4	3	23	38	6
8	CMG Content	9 : CMG Cont	378	81	27	31	13	168	60	85	378	35	43	40	47	30	5	8	33	19	20	25	72	61	42	16	3	5	11	16	71	5	22	29	31	37	106	15	75	36	23	12	15	134	7	10	26	26	12	33	13	4	23	5	82	32	21	8	42	41	21
9	Guidance for at risk patien	10 : Guidance	35	5	1	2	2	13	6	10	35	35	22	2	6	1	0	0	3	2	1	1	15	8	3	2	1	0	1	1	3	1	3	2	2	4	9	3	14	6	4	4	6	22	3	1	1	7	3	7	5	1	5	1	14	6	4	1	8	4	3
10	Inclusivity	11 : Inclusivit	43	3	1	1	1	22	5	11	43	22	43	2	4	1	0	0	3	1	2	0	11	8	2	1	1	0	0	2	5	2	1	0	0	1	9	1	10	5	2	1	4	17	3	0	1	5	2	5	3	0	4	1	12	2	2	1	8	4	4
11	CMG Development	12 : CMG Dev	116	30	11	6	4	30	26	42	40	2	2	116	36	6	2	2	41	13	32	3	46	27	29	8	1	2	7	3	14	1	16	32	27	49	25	3	21	6	8	3	5	24	3	2	8	2	0	6	1	0	5	2	12	5	4	2	5	27	5
12	CMG Evidence Base & Res	13 : CMG Evic	162	20	5	4	0	46	20	32	47	6	4	36	162	18	1	12	22	9	16	21	41	25	12	6	1	2	6	2	14	1	5	15	9	19	27	3	20	8	5	5	5	46	1	2	19	8	2	12	7	0	7	3	30	8	4	3	6	23	8
13	CMG Utilisation by Staff	14 : CMG Util	97	45	15	17	3	55	21	14	30	1	1	6	18	97	21	36	21	15	9	17	30	15	10	9	3	3	5	8	34	4	7	5	13	11	28	10	23	13	6	3	2	23	2	1	12	4	3	5	2	1	3	3	26	8	5	4	6	24	13
14	HCW Lack of CMG Engage	15 : HCW Lac	21	16	10	4	1	16	3	4	5	0	0	2	1	21	21	2	2	2	0	1	5	2	3	3	2	2	1	3	12	1	2	1	4	2	6	2	4	0	3	0	1	2	0	0	0	1	1	0	0	0	0	0	3	2	2	0	1	5	1
15	Monitoring of Utilisation	16 : Monitorin	36	13	3	8	0	17	3	7	8	0	0	2	12	36	2	36	7	7	2	7	6	3	4	3	1	1	2	1	13	1	2	3	2	2	6	3	6	3	2	1	0	10	1	0	2	3	2	0	0	0	0	1	11	3	3	3	0	11	3
16	Local Engagement & Adapt	17 : Local Eng	153	78	17	40	14	43	28	55	33	3	3	41	22	21	2	7	153	95	78	6	43	46	36	10	3	2	7	7	46	2	31	20	42	37	35	6	29	8	14	9	3	32	3	6	12	6	0	8	2	0	7	4	23	9	3	4	18	31	13
17	Local Adaptation of CMGs	18 : Local Ada	95	66	16	35	9	25	17	27	19	2	1	13	9	15	2	7	95	95	20	4	31	20	10	6	3	1	3	4	37	1	21	12	27	26	19	5	20	6	10	7	2	22	2	5	7	6	0	3	2	0	2	1	15	8	3	3	11	11	10
18	Local Engagement of exper	19 : Local Eng	78	18	1	8	7	24	17	36	20	1	2	32	16	9	0	2	78	20	78	3	19	33	28	7	1	1	6	4	15	1	13	12	23	18	19	2	12	2	6	3	1	15	1	2	7	1	0	6	1	0	5	4	13	3	2	2	9	23	5
19	Pos. Impact of CMGS	20 : Pos. Impa	107	33	4	5	9	27	7	11	25	1	0	3	21	17	1	7	6	4	3	107	16	29	11	4	2	0	3	13	20	2	5	3	9	8	45	5	28	15	6	10	1	45	5	3	20	1	4	26	16	3	10	5	18	5	1	2	16	13	8
20	COVID-19	22 : COVID-1	209	111	28	28	24	125	65	48	72	15	11	46	41	30	5	6	43	31	19	16	209	102	29	24	8	6	14	24	63	6	54	37	66	77	61	26	80	37	31	21	9	109	10	29	16	14	6	52	20	10	40	17	63	19	15	7	44	19	34
21	FVDs	23 : FVDs	262	99	22	35	35	85	33	49	61	8	8	27	25	15	2	3	46	20	33	29	102	262	50	26	11	12	9	29	74	10	30	43	43	64	48	21	113	46	56	38	6	69	12	13	15	4	2	57	23	4	44	17	36	7	4	3	47	24	11
22	Expertise & Prior or prof. e	25 : Expertise	196	39	13	29	10	53	7	41	42	3	2	29	12	10	3	4	36	10	28	11	29	50	196	24	7	7	17	15	60	4	22	28	20	29	60	8	38	17	23	8	0	41	2	2	6	4	3	20	3	1	16	5	36	8	6	7	19	26	6
23	HCW Deployment & Colla	26 : HCW De	82	24	4	7	6	29	5	10	16	2	1	8	6	9	3	3	10	6	7	4	24	26	24	82	25	21	51	19	32	2	8	7	13	18	34	7	35	17	19	10	1	23	4	4	8	3	2	6	3	1	2	11	18	9	7	1	17	9	3
24	Availability, Willingness of	27 : Availabili	25	10	2	4	4	15	2	3	3	1	1	1	1	3	2	1	3	3	1	2	8	11	7	25	25	9	4	14	15	2	1	2	5	5	5	3	16	10	10	3	0	5	2	1	0	0	1	2	2	0	0	9	5	3	3	0	12	6	0
25	Deployment of Personnel	28 : Deploym	21	8	2	2	1	8	1	2	5	0	0	2	2	3	2	1	2	1	1	0	6	12	7	21	9	21	3	4	11	0	1	4	5	9	7	4	10	3	8	4	0	5	2	0	2	0	0	0	0	0	0	4	3	0	1	0	5	3	1
26	IP Collaboration	29 : IP Collab	51	9	1	2	2	15	3	7	11	1	0	7	6	5	1	2	7	3	6	3	14	9	17	51	4	3	51	7	13	0	7	4	8	9	27	1	15	8	6	4	1	18	2	3	6	3	2	4	1	1	2	2	14	8	6	1	5	4	2
27	HCW Emotions	30 : HCW Em	110	19	2	6	12	61	5	12	16	1	2	3	2	8	3	1	7	4	4	13	24	29	15	19	14	4	7	110	30	5	2	3	7	5	29	2	35	20	15	8	1	32	5	1	7	0	2	16	10	5	9	8	23	6	4	3	30	5	3
28	HCW Training	31 : HCW Tra	319	138	55	109	13	115	23	35	71	3	5	14	14	34	12	13	46	37	15	20	63	74	60	32	15	11	13	30	319	12	39	25	46	56	101	16	80	33	50	12	2	57	5	16	10	6	5	17	8	1	10	10	51	11	11	7	29	56	9
29	Loss or Infection of HCW	32 : Loss or In	25	15	2	3	2	11	3	5	5	1	2	1	1	4	1	1	2	1	1	2	6	10	4	2	2	0	0	5	12	25	3	0	5	6	2	0	20	16	6	2	1	4	1	2	1	1	0	6	4	0	4	2	7	2	1	0	4	5	1
30	District & Regional	35 : District &	104	46	12	26	7	37	17	24	22	3	1	16	5	7	2	2	31	21	13	5	54	30	22	8	1	1	7	2	39	3	104	18	56	46	25	19	32	13	12	8	6	23	2	23	5	2	0	11	6	2	7	4	25	6	5	3	14	11	5
31	Internat. Partners (e.g., WI	36 : Internat.	106	37	17	12	4	26	16	36	29	2	0	32	15	5	1	3	20	12	12	3	37	43	28	7	2	4	4	3	25	0	18	106	19	67	21	8	27	9	17	6	3	17	2	2	3	2	1	3	0	0	3	3	14	3	2	3	7	7	2
32	Local (rural) level	37 : Local (ru	125	59	16	30	12	42	28	21	31	2	0	27	9	13	4	2	42	27	23	9	66	43	20	13	5	5	8	7	46	5	56	19	125	55	26	16	38	17	16	9	6	24	3	19	6	2	0	10	5	3	7	7	30	3	3	3	21	21	10
33	National level (e.g., MOH)	38 : National	171	76	26	29	16	52	27	35	37	4	1	49	19	11	2	2	37	26	18	8	77	64	29	18	5	9	9	5	56	6	46	67	55	171	34	17	50	18	31	16	4	30	4	15	8	2	3	11	4	0	10	10	22	4	4	5	17	13	9
34	Facilitators & Success	39 : Facilitato	381	100	25	40	21	72	21	55	106	9	9	25	27	28	6	6	35	19	19	45	61	48	60	34	5	7	27	29	101	2	25	21	26	34	381	8	77	30	41	24	5	97	4	10	32	13	9	33	15	4	21	6	72	20	14	12	48	46	8
35	HealthCare System Issues	40 : HealthCa	57	15	4	6	5	39	12	9	15	3	1	3	3	10	2	3	6	5	2	5	26	21	8	7	3	4	1	2	16	0	19	8	16	17	8	57	26	8	16	10	0	18	1	12	6	1	0	8	5	1	4	7	23	6	3	0	10	12	6
36	Pandemic Preparedness &	41 : Pandemi	276	86	16	26	38	113	18	53	75	14	10	21	20	23	4	6	29	20	12	28	80	113	38	35	16	10	15	35	80	20	32	27	38	50	77	26	276	110	132	80	21	58	15	20	18	6	4	51	22	6	35	16	56	12	9	4	50	43	14
37	Infection Prevention & Co	42 : IPC & He	110	31	2	9	13	46	12	21	36	6	5	6	8	13	0	3	8	6	2	15	37	46	17	17	10	3	8	20	33	16	13	9	17	18	30	8	110	110	21	18	4	21	7	7	12	1	2	28	13	3	18	7	34	6	4	1	24	21	9
38	Preparedness	43 : Prepared	132	42	10	15	18	53	3	17	23	4	2	8	5	6	3	2	14	10	6	6	31	56	23	19	10	8	6	15	50	6	12	17	16	31	41	16	132	21	132	21	0	24	3	6	4	0	1	16	7	2	11	9	21	3	3	1	22	26	3
39	Testing, Surveillance & Ear	44 : Testing, S	80	23	3	4	20	25	4	8	12	4	1	3	5	3	0	1	9	7	3	10	21	38	8	10	3	4	4	8	12	2	8	6	9	16	24	10	80	18	21	80	2	22	10	10	7	4	1	17	6	2	15	6	15	4	5	3	20	7	4
40	Vaccination	45 : Vaccinat	21	10	3	3	1	13	0	11	15	6	4	5	5	2	1	0	3	2	1	1	9	6	0	1	0	0	1	1	2	1	6	3	6	4	5	0	21	4	0	2	21	4	0	0	0	3	1	5	3	1	4	0	7	3	1	1	4	2	1
41	Patient Care & Standardis	46 : Patient C	360	70	11	19	15	127	33	52	134	22	17	24	46	23	2	10	32	22	15	45	109	69	41	23	5	5	18	32	57	4	23	17	24	30	97	18	58	21	24	22	4	360	28	28	71	41	25	22	8	5	15	15	61	42	21	7	50	21	16
42	Psycho-social Treatment	47 : Psycho-s	28	8	1	1	3	10	2	4	7	3	3	3	1	2	0	1	3	2	1	5	10	12	2	4	2	2	2	5	5	1	2	2	3	4	4	1	15	7	3	10	0	28	28	2	3	0	0	3	3	0	3	4	5	1	0	0	10	5	3
43	Referral & Transfer of Patie	48 : Referral &	52	14	0	6	1	27	8	1	10	1	0	2	2	1	0	0	6	5	2	3	29	13	2	4	1	0	3	1	16	2	23	2	19	15	10	12	20	7	6	10	0	28	2	52	2	0	4	12	4	4	6	2	25	12	8	1	4	3	3
44	Standardised Care	49 : Standard	71	26	1	5	3	19	6	17	26	1	1	8	19	12	0	2	12	7	7	20	16	15	6	8	0	2	6	7	10	1	5	3	6	8	32	6	18	12	4	7	0	71	3	2	71	0	0	4	2	0	3	3	13	2	2	1	7	8	6
45	Therapeutics	50 : Therapeu	41	9	2	2	1	17	2	15	26	7	5	2	8	4	1	3	6	6	1	1	14	4	4	3	0	0	3	0	6	1	2	2	2	2	13	1	6	1	0	4	3	41	0	0	0	41	5	7	3	0	5	0	24	23	9	2	2	2	6
46	Treament (IV lines, dozing,	51 : Treament	25	2	0	3	0	18	1	2	12	3	2	0	2	3	1	2	0	0	0	4	6	2	3	2	1	0	2	2	5	0	0	1	0	3	9	0	4	2	1	1	1	25	0	4	0	5	25	7	2	3	4	0	21	18	9	4	2	0	4
47	Patient Outcomes (Mortal	52 : Patient O	135	16	0	6	10	47	7	19	33	7	5	6	12	5	0	0	8	3	6	26	52	57	20	6	2	0	4	16	17	6	11	3	10	11	33	8	51	28	16	17	5	22	3	12	4	7	7	135	51	22	91	6	31	13	8	3	24	10	15
48	Mortality	53 : Mortality	51	7	0	2	4	17	4	6	13	5	3	1	7	2	0	0	2	2	1	16	20	23	3	3	2	0	1	10	8	4	6	0	5	4	15	5	22	13	7	6	3	8	3	4	2	3	2	51	51	4	19	5	12	2	3	2	10	5	10
49	Patient Volume	54 : Patient V	22	1	0	1	1	9	0	1	4	1	0	0	0	1	0	0	0	0	0	3	10	4	1	1	0	0	1	5	1	0	2	0	3	0	4	1	6	3	2	2	1	5	0	4	0	0	3	22	4	22	8	1	8	6	3	1	4	1	0
50	Symptom Severity & Chan	55 : Symptom	91	9	0	3	7	28	3	13	23	5	4	5	7	3	0	0	7	2	5	10	40	44	16	2	0	0	2	9	10	4	7	3	7	10	21	4	35	18	11	15	4	15	3	6	3	5	4	91	19	8	91	3	20	8	3	2	19	6	7
51	Political & Economic Issue	56 : Political &	41	14	2	1	15	25	4	7	5	1	1	2	3	3	0	1	4	1	4	5	17	17	5	11	9	4	2	8	10	2	4	3	7	10	6	7	16	7	9	6	0	15	4	2	3	0	0	6	5	1	3	41	11	0	0	1	28	3	1
52	Resourcing (e.g., Therapeu	57 : Resourci	234	38	8	22	11	145	31	36	82	14	12	12	30	26	3	11	23	15	13	18	63	36	36	18	5	3	14	23	51	7	25	14	30	22	72	23	56	34	21	15	7	61	5	25	13	24	21	31	12	8	20	11	234	73	41	28	34	36	24
53	Drugs-supplies-infrustruct	58 : Drugs-su	73	14	3	8	1	46	7	7	32	6	2	5	8	8	2	3	9	8	3	5	19	7	8	9	3	0	8	6	11	2	6	3	3	4	20	6	12	6	3	4	3	42	1	12	2	23	18	13	2	6	8	0	73	73	33	5	7	6	8
54	Equipment & supplies (plu	59 : Equipme	46	4	2	5	1	31	6	4	21	4	2	4	4	5	2	3	3	3	2	1	15	4	6	7	3	1	6	4	11	1	5	2	3	4	14	3	9	4	3	5	1	21	0	8	2	9	9	8	3	3	3	0	41	33	46	9	6	5	4
55	Funding	60 : Funding	28	4	1	7	1	17	3	3	8	1	1	2	3	4	0	3	4	3	2	2	7	3	7	1	0	0	1	3	7	0	3	3	3	5	12	0	4	1	1	3	1	7	0	1	1	2	4	3	2	1	2	1	28	5	9	28	3	4	1
56	Social & Societal Issues	61 : Social & S	169	37	5	7	51	62	14	23	42	8	8	5	6	6	1	0	18	11	9	16	44	47	19	17	12	5	5	30	29	4	14	7	21	17	48	10	50	24	22	20	4	50	10	4	7	2	2	24	10	4	19	28	34	7	6	3	169	21	2
57	Suggestions	62 : Suggestic	135	48	11	19	7	59	16	38	41	4	4	27	23	24	5	11	31	11	23	13	19	24	26	9	6	3	4	5	56	5	11	7	21	13	46	12	43	21	26	7	2	21	5	3	8	2	0	10	5	1	6	3	36	6	5	4	21	135	8
58	Treatment Improvisation	63 : Treatmen	64	18	5	2	0	40	16	6	21	3	4	5	8	13	1	3	13	10	5	8	34	11	6	3	0	1	2	3	9	1	5	2	10	9	8	6	14	9	3	4	1	16	3	3	6	6	4	15	10	0	7	1	24	8	4	1	2	8	64

Lift Filter applied to the Matrix = (Occurrence of Code A/ Co-Occurrence of A and B)/(Occurrence of Code B/Total Occurences in Network)

$$\text{Lift} = \frac{\left(\frac{\text{Occurrences of Code A}}{\text{Co-occurrences of A and B}}\right)}{\left(\frac{\text{Occurrences of Code B}}{\text{Total Occurrences in Network}}\right)}$$
