# Supplementary material for: ‘Involve those who are managing these outbreaks’: stakeholders’ perspectives on the barriers and facilitators to the implementation of clinical management guidelines for high-consequence infectious diseases in Uganda—a thematic network analysis
Source: BMJ Public Health. 2025 Feb 13;3(1):e001165. doi: 10.1136/bmjph-2024-001165 (PMC11843484; doi:10.1136/bmjph-2024-001165)
Supplement: online supplemental file 5 [file bmjph-3-1-s005.pdf]

Thematic Data Extraction Example Table			
**Example References for Cluster**	**Interpretation of Reference**	**Thematic Codes applied to reference**	**Thematic Clusters derived through modularity algorithm***
For Marburg there was little apart from checking the internet, so […] when we were confronted […] I based [treatment] on the old knowledge [from] seven years earlier (MP19).	Availability of CMGs patchy and outdated, frequently occurring & prioritised HCIDs being prioritised over less frequent HCIDs.	Barriers & Challenges, Access & Dissemination of Information, Dissemination by offical Communication	
One [barrier] is human resource, if people are many, guidelines can be followed to the dot but if people are few, certain things are forgotten. […] two, lack of knowledge, people are not fully trained and if trainings are not practical, they will never get skills. But also, three, lack of guidelines, they might be there but someone has never seen them. Even the new guidelines of Zaire strain, I don't have. Another thing is poor reading culture. In Uganda, we don't read (MP21).	Lack of staff,inadequate training, poor dissemination, poor reading culture as main barriers to implementation of CMGs	Barriers & Challenges, Access & Dissemination, HCW training, Resourcing (Equipment, Therapeutics, Staff), CMG Utilisation by staff,	
I think it could even take two months in a place like Fort Portal, you could find when they have updated the guidelines but they have not yet shared them and all of a sudden you come and land on the book about the new guideline on the internet by chance (MP7).	Slow and patchy dissemination in rural aread,HCP unaware of guidelines, describing challenges accessing guidelines, limited announcements.	Barriers & Challenges, Access & Dissemination of Information, CMG Change & Updates, Dissemination by offical Communication	

Those initial technical working groups were composed of experts, from the Ministry of Health, from other stake holders, WHO, CDC academia and also programmatic partners. So, that sat together to initiate this process of drafting the guidelines. And of course, subsequently it was presented to the various pillars and the pillars also had sub pillars which also picked at various components. We had particularly the case management were I was, I think we had the clinical sub pillar the IPC pillar and the mental health pillar. I think those were the important components. So, we further scrutinized those pillars, updated and discussed in the	CMG development pathway, alongside national and international partners, representing different sub pillars.	CMG Development & Updates, national level, international partners	**CMG Dissemination & Training (representing 30.36% of Graph)**
There should be practical sessions and continuous follow up of training outcomes [...] if you just come for a week and train people, let's say on how to manage EVD, tomorrow if someone is faced with a patient, they will fail to manage electrolyte balance. Not because they don't know, but because they did not have a practical session, and no continuous mentorship (MP4).	Adherence to guidance due to lack of practical training with continued mentorship at the healthcare facility.	Faciliators & Success, HCW training, CMG Content, Pandemic Preparedness	
If you want to improve the standard and I think this is the most important rule of research, if we see that apparently this patient is receiving x for their care, can we do X plus 1 and x plus 2 and then compare which of the two patients do better. But now we have once we have those guidelines we will be able to see how we raise the standards higher by showing evidence that when we go beyond research and change the guidelines to get a better outcome. (CM1)	Changing of standards and CMGs for Patient Care depends on adequate Evidence base & research	Patient Care & Standardisation, CMG Evidence Base & Research, Positive Impact of CMGs, CMG, Change & Updates of guidelines, Treatment Improvisation	
Our local teams [need to be] involved because they are the ones who are managing these outbreaks (CM3). If we are to have guidelines which can be useful, then we should tailor them to the local context of what is happening in Uganda (MP4)	Input of local stakeholders able to adapt guidelines to available local resources	Facilitators & Success, Dirstrict & Regional, Logal Engagement & Adaptation, CMG Development	

[In] lower-level facilities […] you rarely get the Filovirus guidelines […]. Dissemination is really a big problem, [especially as] the common venue where we have these outbreaks is more in the rural areas where the human being interfaces with the wild population and that's where you're going to get the lowest level of health centres. We tend to concentrate at the big facility level while this should be going down there, and from down there it goes upwards (TM4).	CMGs for FVDs rare in lower level facilities, detrimental to pandemic response as outbreaks tend to first occur in such rural areas.	Barriers & Challenges, Access & Dissemination of Information, Pandemic Preparedness & Response, District & Regional Level, National Level
A case definition of an epidemic is developed from that particular epidemic. You don't develop it that this is a book definition […]. No, you must look at the patient […] and keep revising the case definition […] That's why they used to call it the viral Heamorrghic fever because [patients were] either bleeding, or passing out blood in stool or vomiting blood but [with] West African Ebola, we could not see much and that's why we [now] call it Ebola Virus Disease (EVD) (TM1).	As the guidance for Viral Haemorrhagic Fever focused on bleeding as a main symptom, so the case definition for West African Ebola which did not cause such symptoms was updated.	CMG Change & Updates, CMG Content, Patient Outcomes, Symptom Severity, & Change, FVD
We had several avenues for doing this, we had the guidelines that were produced in print, and the books were[then] distributed across the country. But before the distribution happened, we made sure that we conducted a series of orientations to all health workers. Well we had Training of Trainers conducted at national level and as a way of enhancing penetration and building capacity at regional level, we co-opted that the regional coordinators of all Regional hospitals into the national trainers (CM4).	Hard copies of guidelines delivered alongside cascading training at different levels of healthcare system.	Access & Dissemination of Information, HCW training, Dissemination by Training, National level, District & Regional Level, Availability & Access to CMGs, Facilitators & Success
When [CMGs are] disseminated [down], we also try to go to work with the hospitals within this framework what has been disseminated. They [CMGs] can say jik 1.025 but you [only] have soap. Yes, you can use the soap but the whole thing is; they're telling you to wash your hands. So you improvise, if its soap that is there you use soap, if they say jik and there is no jik, you have chlorine powder, you mix the chlorine and have a disinfectant (MP21).	Content of CMGs needs to be adapted at the local level and people trained in the adaptation of the CMGs based on their available resources.	CMG Applicability to Setting, Patient Care & Treatment, HCW training, Local Engagement & Adaptation, Treatment Improvisation

Let them be accessible.[…] I need to have mine as a person, not to have them somewhere, [and] you wait until the problem comes then you go and look for them.[if]we go there and say 'doctor can we have the recent guideline on Covid' it will take him two days to get it. Or he is also going to call me back and say xx where do you think we put this guideline. T[…] They need to be if possible let every clinician, nursing team, library, DHO's office have guidelines. Let them be there as our daily food (MP5).	CMGs need to be immediately accesible and widely distributed to every HCW.	Access & Dissemination of Information, Barriers & Challenges, Pandemic Preparedness	
The WHO has been using […] the living guidelines approach rather than updating the guidelines every 6 to 9 months or so we update whenever we have information but we have to update, disseminate and train. It is easier if we know that the guidelines are stable and there are many studies that might be happening that can cause the guidelines to change, then it is easier to have one guideline revision that applies to all the theurapeticals (CM1).	Living guidance as best case, but training is needed and	CMG Content, Patient Care & Standardisation, HCW Training, CMG Change & Updates of guidelines, Facilitators & Success, COVID-19	
In [the] West Africa [outbreak], we tried to develop guidelines for children. […] but the guidelines we were using were not sufficient to cover vulnerable groups especially children, pregnant women, elderly and people with disabilities. So, we were just cramping everyone together (MP9).	CMGs not equally inclusive of patient groups (e.g., HIV/immunocompromised, pregnant women, children, elderly) and not always updated accordingly	CMG Content, Barriers & Challenges, Inclusivit, FVD	
Then the special groups, a disease has a kind of behavior to a certain group of people. So it should be very clearly stipulated how best we could manage these people, for example if the disease behaves differently on someone with HIV, […] that should be very important for the guidelines to stipulate, when you have a patient of Covid-19 with HIV, this is how you're supposed to manage them. So I think these are some of the things they might not have covered fully but they were very critical in these guidelines. (MP4)	CMGs not equally inclusive of patient groups (e.g., HIV/immunocompromised, pregnant women, children, elderly) and not always updated accordingly	CMG Content, Barriers & Challenges, Inclusivity, COVID-19	

When it comes to urban versus rural though, there are some things that apply in urban setting that may not apply in the rural setting, e.g., a patient who develops a complication while at home, can easily be evacuated to the facility in an urban setting, but in the rural setting, means of transport are so scarce that it may not work there (MP2).	Urban/ rural divide in the healthcare system makes adequate Patient care difficult, CMG content needs to reflect this and needs to be applicable in all settings.	CMG Content, Applicability to Setting, Resourcing (Therapeutics, Equipment, Staff), Patient Care & Standardisation, Barriers & Challenges	**CMG Content & Updates (representing 25% of graph)**
Implementation is a different thing [as] the health workers knew what to do, [if] they didn't do, it was because of lack of adequate infrastructure; particularly for critical care, [it was] an issue both in terms of human expertise and also infrastructure" (CM6).	The intersection between access to guidelines, lack of resources and HCP's ability to adhere to guidelines was most evocative in this reflection:	CMG Utilisation, Resourcing (therapeutics, Equipment, Staff), HealthCare System Issues, Barriers & Challenges	
Having guidelines [is one thing] but having the resources to buy what the guidelines recommend is another one. If the guideline said give out oxygen and oxygen was not there, then it will be difficult.[…] There was an issue of use of profolactive drug for preventing clot formation. This is very expensive. So, we found a lot of variations at both sides, some are using it and some are not using the drug that prevents the clot just because of lack of access. (TM2).	Guidelines did not represent the available resources, especially therapeutics, available.	CMG Content, Applicability to Setting, Resourcing (Therapeutics, Equipment, Staff), Treatment Improvisation, Patient Care & Standardisation, Barriers & Challenges	
In the private sector, there was a lot of resistance […] we discovered that there were some [expensive] drugs that had not been recommended to treat COVID 19, but they were using it and not adhering to our guidelines […]. We also had instances where clinics were reusing PPE, coveralls which were supposed to be single uses […they] sprayed it with chlorine on the outside and put it out to dry and then reused it (TM2).	Implementation of CMGs inhibited by differences between private and public healthcare facilities, where private facilities may distribute therapeutics more quickly or instate different infection prevention and control (IPC) measures.	CMG Content, COVID-19, Healthcare System Issues, Treatment Improvisation, Patient Care & Standardisation	

It's not only for Covid-19, we have other guidelines like our Uganda CMGs [but]unfortunately that system is not followed, [because] we already have no proper regulation. So getting guidelines for new diseases is not necessarily going to get monitored because the system is not functional. We need to functionalize the whole system from […] the health unit being registered […] to the supplier knowing that they're not supposed. But with the very developed private practice, we have lost control to regulate (CM5).	Implementation of CMGs inhibited by lack of monitoring and differences between private and public healthcare facilities.	CMG utilisation by Staff, Barriers & Challenges, Resourcing, (e.g., Therapeutics, Equipment, Staff), Patient Care & Standardisation, Healthcare system Issues, COVID-19	
It has been a blended approach of physical, virtual, whatsApp groups providing updates, emails. But it is worth evaluating how far they reached because […] it would be good to know how these lower levels are managing Covid. They adequately received the information, however, […] they will tell you how they have never been trained, yet they have been trained [but] they don't attend. [or] they don't read. So one thing I have noted with these lower facilities you check whatever they are saying with attention given those limitations. CM3	Implementation of CMGs impaired by lack of engagement with guidelines, thus monitoring of utilisaton required.	CMG Utilisation by Staff, Monitoring of Utlisation, Barriers & Challenges, Access & Dissemination, HCW training, COVID-19	
It affects the who continuum of life …] we have had destruction of health care delivery and this has caused several challenges that have resulted from the outbreak. Recently we had essential services disrupted especially those for managing chronic diseases - going without medications. We also had several preventable deaths […] and had limited access to care for the mothers and we also had preventable deaths of the mothers and neonates or children. The impact was social and economical (CM2)	Discussing how disruption of essential services for disease was impacted by the outbreak, leading to adverse patient outcomes.	Patient Outcomes, Barriers & Challenges, Social & Societal Issues, Political 7 Economic Issues, Mortality	
They said 'Museveni had brought a disease' , 'These people are lying to us'. 'They want to take us to the hospital and kill you from there'. That was the situation around here, so we had to stop […] burying people who were positive. We had to withdraw because [if] you bury someone they want to beat you, they see you taking the dead body. They say 'you have killed our relative because you want money. The government is giving you people money to kill our relatives'. So that was the biggest problem we got from the community (MP3).	impeded their ability to implement guidelines and carry out their job according to guidelines.	Patient Care & Standardisation, Social & Societal Issues, Political & Economic Issues, HCW Emotions, HCW Deployement & Teamwork, COVID-19	

Before training, we where really very worried; I was worried. But having gained the knowledge and the skills and experience of seeing others working on the patients, my level of confidence increased. We were even assured during the training that we are much better because for us we are dealing with patients whom we are suspecting already, and so it makes our level of suspicion and our level of alertness being high. (MP22).	Training was reported to increase confidence and self-efficacy, suggesting that this would lead to better implementation of CMGs,	HCW Emotions, Facilitators & Success, HCW Training, Patient Care & Standardisation, Positive Impact of CMGs	**Patient Care Outcomes & Standardisation (representing 26.79% of graph)**
Patients that are managed according to protocols and guidelines have a better chance of survival and are being discharged to go home, [and] the more people that go back home, the more the community trust[s] us and the earlier the patients – if they […] get VHF – will come to treatment centres for testing and further management. (MP20)	Lack of resources was recurrently associated with increased infection risk and mortality such as in the following quote	Patient Care & Standardisation, Patient Outcomes, Social & Societal Issues, Positive Impact of CMGs	
The challenge was asking for services that were not possible at the different levels of care. A severely ill patient would be managed at the general hospital where they require high dependence support and the regional hospital does not have the high dependence support care at the regional referral level. In such a case, you find yourself referring the patient and when that happens, there is an automatic delay in diagnosis and treatment of the patient and that automatically affected the outcome of the patient. (CM2)	Patient Care being impacted by different availability of diagnostics and care at different levels of thehealthcare system.	Patient Care & Standardisation, Resourcing (e.g., Therapeutics, Equipment, Staff), District & Regional, Equipment & Supplies, HealthCare System Issues	
[Because of] religion and cultural beliefs, you find the community refusing that there is Ebola, they refuse to come to hospital, they report late [and] health workers also believe that their religion doesn't allow them to perform some rituals (TM3).	Lack of adherence to Public Health guidance by the population, which hinders the effective response to outbreaks an implementation of CMGs	Social & Societal Issues, Patient Care & Standardisation, Patient Outcomes, Pandemic Preparedness & Response, FVD	

You just see something passing on social media that there is someone who died of Ebola 'be careful'. I think we need to be informed and updated on whatever is going on. Now if there is a case in Congo it means Congo is just here the next patient will be here because we have a lot of patients who cross [the border] to come here for services. […]the second case was in the medical division and it was from Congo and they […] passed through porous borders. […] so we need to be prepared all the time. (MP3)	Discussion of the necessity to have adequate cross-border response to HCID outbreaks.	Pandemic Preparedness & Response, District & Regional, Local (rural)level, Public Health Messaging & Implementation	**Pandemic Preparedness & Response (representing 10.71% of graph)**
I […] found a nurse drawing blood with bare hands, no gloves [though] on the trolley were gloves, [so…] we have these guidelines but for some reasons they are not being followed [and] that's why health workers in ETU and CTUs die. (TM5).	Discussion of the negative consequences for IPC and loss of colleagues due to Inadequate adherence of CMGs	Pandemic Preparedness & Response,IPC & Health & Safety, Loss or Infection of colleagues, Resourcing (Therapeutics, Equipment, Staff), CMG Utilisation by Staff	
I think using surveillance which is related to early identification of these cases has really helped. I don't remember if there was even a transmission beyond that one case. You know it has been 3 years. I don't remember if we had other than that mother and the child. I don't think we had a transmission beyond that (CM3)	Surveillance discussed as related to effective outbreak response and control.	Patient Outcomes, Pandemic Preparedness, Testing, Surveillance & Early Warning, Facilitator & Success	
If the person doesn't have masks, they won't be safe, […if] they go and operate an Ebola case – like what happened in Mubende [in Nov 2022] – then they get infected (TM3).	Lack of resource associated with increased infection risk and mortality such as in the following quote	Pandemic Preparedness & Response, District & Regional, IPC & Health & Safety, Loss or Infection of colleagues, FVD	

The Mental health [pillar] is not given a lot of opportunity to participate. We come when things are not working very well; they call in these guys of psychosocial support when everyone is stressed. The challenges that we have had is that health workers tend to run away from the facilities, when there is an epidemic. […] But there are those who come in much later, and sometimes our people; the mental health providers come in later, and […] we are not including them immediately, they also run away and only come back when they are comfortable a little later when they are sure that chances of dying of the infection are reduced	Ineffective inter-professional collaboration and inclusion limits the effective pandemic response and effects the wilingness of staff to work	Healthcare Worker Deployment & Teamwork Patient Care & Standardisation, Social and Societal Issues,HCW training, Deployment of personnel, Bariers and Challenges,	**Healthcare Teamwork and Deployment (representing 7.14% of graph)**
Updates would be for everyone, the case management team, the health workers who are not part of the case management, the village health teams, even the community to create awareness. If everybody is aware and such an outbreak comes again, everybody would be on standby and it wouldn't take us or kill us the way it did previously (MP5).	Effective inter-professional collaboration and depends on dissemination of CMGs (and their updates) to every pillar and segment within the healthcare system.	Healthcare Worker Deployment & Teamwork Patient Care & Standardisation, Facilitators, CMG Access & Disemination	
The guideline indicated that all the patient's property; […] everything that this person touched should be burned , apart from the house. The house is cleaned but every other thing you get them and burn them you get, but [we're] not replacing these things. The health workers who do that are on the risk of being stoned and even they can kill you. Because you are going to burn someone's property but you are not replacing them. So, some of these things are not well spelled in that guideline. […]You know we are serving the living God and we say above all we can do no harm […] but no one declares that I shall always risk my life. (MP-14)	Inadeqaute discussions of the societal and Politial Issues that limit effective CMG implementation effect the willingness of staff to work.	Healthcare Worker Deployment & Teamwork, Willingness, Barriers & Challenges, Political & Economic Issues, Social & Societal Issues	
We would also realize that there was a time when there was a burn out. Very heavy workload, very many patients, these also contributed to either adhering more or less adherence to the guidelines because if someone is very tired and they're faced by these patients and they don't know what to do because they're very tired so you will find that there is a breach protocol, the breach of maybe PPE and all that were happening during the management of these patients. And then other factors like frequent and continuous reminders, workshops, trainings, and all those fostered adherence to the guidelines (MP 4)	Effect of long-term deployment on staff-members impacts Implementation and adherence of CMGs leading to improvisations and breaches of SOPs, but can be impacted by frequent training.	Healthcare Worker Deployment & Teamwork Social & Societal Issues, HCW Emotions, Barriers & Challenges, CMG Utilisation by Staff, Treatment Improvisation, HCW training	

*The data was filtered using a Lift filter (Lift < 1), which filtered out any associations which were likely to have occurred by chance. A Leiden algorithm for cluster detection was used to identify thematic clusters in the graph, showing moderate modularity (Modularity = 0.362) with 5 clusters representing themes that show more connections with each other than with
