# Supplementary material for: ‘Involve those who are managing these outbreaks’: stakeholders’ perspectives on the barriers and facilitators to the implementation of clinical management guidelines for high-consequence infectious diseases in Uganda—a thematic network analysis
Source: BMJ Public Health. 2025 Feb 13;3(1):e001165. doi: 10.1136/bmjph-2024-001165 (PMC11843484; doi:10.1136/bmjph-2024-001165)
Supplement: online supplemental file 6 [file bmjph-3-1-s006.pdf]

Clustertable

This table shows all codes within their respective thematic clusters alongside the number of references each code appear in, the average degree, average weighted degree and betweenness centrality. The codes are sorted by their weighted degree, highlighting the importance of the code as a function of the number of connections between this code and other codes within the network.

Thematic Code Label	Total References	Degree	Weighted Degree	Between-ness Centrality	Cluster	Cluster Label
Access & Dissemination of Information	396	14	1681	109.98	1	CMG Development & Dissemination (representing 30.36% of Graph)
Facilitators & Success	381	6	1262	2.77	1	
HCW Training	319	14	1249	18.87	1	
CMG Change & Updates of guidelines	254	8	962	5.58	1	
Local Engagement & Adaptation	153	17	944	118.76	1	
National level (e.g., MOH)	171	17	862	22.77	1	
Expertise & Prior or prof. experience	196	12	751	7.17	1	
Local (rural) level	125	20	717	31.04	1	
Suggestions	135	21	680	74.98	1	
District & Regional	104	17	580	27.75	1	
CMG Development	116	12	567	14.96	1	
CMG Evidence Base & Research	162	7	523	2.55	1	
Internat. Partners (e.g., WHO, NGOs)	106	13	499	18.05	1	
Dissemination by official Comms	115	2	139	0.00	1	
Dissemination by Training	133	2	124	0.00	1	
Local Adaptation of CMGs	95	2	115	0.00	1	
Local Engagement of experienced staff	78	2	98	0.00	1	
Barriers & Challenges	511	11	1855	9.66	2	CMG apllicability to patients, setting, and resources (representing 25% of graph)
CMG Content	378	11	1483	133.35	2	
Resourcing (e.g., Therapeutics, Equipment, Staff)	234	20	1168	196.95	2	
CMG Applicability to Setting	110	18	588	85.83	2	
CMG Utilisation by Staff	97	18	549	146.76	2	

HealthCare System Issues	57	19	333	68.56	2	
Treatment Improvisation	64	12	290	28.22	2	
Drugs-supplies-infrastructure	73	3	111	0.00	2	
Equipment & supplies (plus infrastructure and Transport)	46	3	83	0.00	2	
Inclusivity	43	2	65	0.00	2	
Guidance for at risk patient groups	35	2	57	0.00	2	
Funding	28	3	42	0.00	2	
Monitoring of Utilisation	36	2	38	0.00	2	
HCW Lack of CMG Engagement	21	2	23	0.00	2	
Patient Care & Standardisation	360	12	1403	259.29	3	Patient Care Outcomes & Standardisation (representing 26.79% of graph)
Social & Societal Issues	169	13	705	32.71	3	
Patient Outcomes (Mortality, Severity, Volume)	135	13	653	158.57	3	
Pos. Impact of CMGS	107	13	443	48.98	3	
HCW Emotions	110	12	427	35.51	3	
Public Health Messaging & Implementation	83	14	402	29.18	3	
Political & Economic Issues	41	17	240	63.85	3	
Symptom Severity & Change	91	3	118	0.00	3	
Standardised Care	71	2	76	0.00	3	
Mortality	51	3	74	0.00	3	
Therapeutics	41	2	46	0.00	3	
Referral & Transfer of Patients	52	3	36	0.50	3	
Treatment (IV lines, dozing, cleaning)	25	3	34	0.50	3	
Patient Volume	22	3	34	0.00	3	
Psycho-social Treatment	28	3	33	0.50	3	
Pandemic Preparedness & Response (e.g.,	276	22	1593	227.11	4	Pandemic Preparedness & Response

Surveillance, Vaccinations)						(representing 10.71% of graph)
Preparedness	132	3	174	0.00	4	
Infection Prevention & Control	110	4	153	0.33	4	
Loss or Infection of HCW	25	18	139	36.92	4	
Testing, Surveillance & Early Warning	80	4	121	0.33	4	
Vaccination	21	3	27	0.00	4	
HCW Deployment & Collaboration	82	21	510	177.12	5	Workforce Collaboration & Engagement (representing 7.14% of graph)
IP Collaboration	51	3	58	0.00	5	
Availability, Willingness of Staff	25	3	38	0.00	5	
Deployment of Personnel	21	3	33	0.00	5	

*The data was filtered using a Lift filter (Lift < 1), which filtered out any associations which were likely to have occurred by chance. A Leiden algorithm for cluster detection was used to identify thematic clusters in the graph, showing moderate modularity (Modularity = 0.362) with 5 clusters representing themes that show more connections with each other than with the rest of the graph.
