# Supplementary material for: ‘Involve those who are managing these outbreaks’: stakeholders’ perspectives on the barriers and facilitators to the implementation of clinical management guidelines for high-consequence infectious diseases in Uganda—a thematic network analysis
Source: BMJ Public Health. 2025 Feb 13;3(1):e001165. doi: 10.1136/bmjph-2024-001165 (PMC11843484; doi:10.1136/bmjph-2024-001165)
Supplement: online supplemental file 8 [file bmjph-3-1-s008.pdf]

Overview of HCID outbreaks in Uganda

Year	Cases	Deaths	Case fatality ratios	High consequence infectious disease
**2000**	425	224	53%	Sudan virus disease
**2007**	149	37	25%	Bundibugyo virus disease
**2012**	06	03	50%	Sudan virus disease
**2012**	07	004	36%	Sudan virus disease
**2007-2018**	23	09	39%	Marburg virus disease
**2020-2023**	171 871	3,632	2%	COVID-19
**2022-2023**	142	55	39%	Sudan virus disease

Data from:

Roddy P. A Call to Action to Enhance Filovirus Disease Outbreak Preparedness and Response. *Viruses*. 2014;6:3699–718. doi: 10.3390/v6103699

WHO World Health Organization. WHO Health Emergency Dashboard - Uganda Situation. 2023. https://covid19.who.int/region/afro/country/ug (accessed 30 November 2023)

Mbonye AK, Sekamatte M. Disease outbreaks and reporting in Uganda. *The Lancet*. 2018;392.

National Emerging Special Pathogens Training and Education Center (NETEC). Situation Report: Uganda Confirmed an Outbreak of a Rare Species of Ebola. 2022. https://netec.org/2022/09/22/situation-report-uganda-confirmed-an-outbreak-of-a-rare-species-of-ebola/ (accessed 30 November 2023)
