# Supplementary material for: ‘Involve those who are managing these outbreaks’: stakeholders’ perspectives on the barriers and facilitators to the implementation of clinical management guidelines for high-consequence infectious diseases in Uganda—a thematic network analysis
Source: BMJ Public Health. 2025 Feb 13;3(1):e001165. doi: 10.1136/bmjph-2024-001165 (PMC11843484; doi:10.1136/bmjph-2024-001165)
Supplement: online supplemental file 9 [file bmjph-3-1-s009.pdf]

Supplementary File 9

Overview of most frequently occurring barriers to CMG implementation in Figure 3, showing total references of the code sorted by the weighted degree in relation to barriers & challenges.

**Themes most commonly related to barriers & challenges**	**Total Ref#**	**Weighted Degree**	**Cluster**
CMG Content	378	168	CMG applicability to patients, settings & resou[illegible]
Resourcing (e.g., therapeutics, equipment, staff)	234	145	CMG applicability to patients, settings & resources
Access & Dissemination of Information	396	139	CMG Development & Dissemination
Patient Care & Standardisation	360	127	Patient Care & Standardisation
HCW Training	319	115	CMG Development & Dissemination
Pandemic Preparedness & Response (e.g., surveillance, vaccinations)	276	113	Pandemic Preparedness & Response
CMG Change & Updates	254	102	CMG Development & Dissemination
Social & Societal Issues	169	62	Patient Care & Standardisation
HCW Emotions	110	61	Patient Care & Standardisation
Suggestions	135	59	CMG Development & Dissemination
CMG Applicability to Setting	110	59	CMG applicability to patients, settings & resources
CMG Utilisation by Staff	97	55	CMG applicability to patients, settings & resources
Patient Outcomes (e.g., mortality, severity, volume of patients)	135	47	Patient Care & Standardisation
Local (rural) level	125	42	CMG Development & Dissemination
Treatment Improvisation	64	40	CMG applicability to patients, settings & resources
