# Supplementary material for: ‘Involve those who are managing these outbreaks’: stakeholders’ perspectives on the barriers and facilitators to the implementation of clinical management guidelines for high-consequence infectious diseases in Uganda—a thematic network analysis
Source: BMJ Public Health. 2025 Feb 13;3(1):e001165. doi: 10.1136/bmjph-2024-001165 (PMC11843484; doi:10.1136/bmjph-2024-001165)
Supplement: online supplemental file 10 [file bmjph-3-1-s010.pdf]

Supplementary File 10

Overview of most frequently occurring suggestions and facilitators for CMG implementation in Figure 4, showing total references of the code sorted by the weighted degree in relation to suggestions and facilitators.

**Themes most commonly related to Facilitators & Suggestions**	**Total Ref#**	**Weighted Degree**	**Cluster**
HCW Training	319	157	CMG Development & Dissemination
Resourcing (e.g., therapeutics, equipment, staff)	234	108	CMG applicability to patients, settings & resources
Expertise & Prior or Professional Experience	196	86	CMG Development & Dissemination
Access & Dissemination of Information	396	48	CMG Development & Dissemination
Positive Impact of CMGs	107	45	Patient Care & Standardisation
Pandemic Preparedness & Response (e.g., surveillance, vaccinations)	276	43	Pandemic Preparedness & Response
CMG Content	378	41	CMG applicability to patients, settings & resources
CMG Change & Updates	254	38	CMG Development & Dissemination
HCW Deployment & Collaboration	82	34	Workforce Collaboration & Engagement
Local Engagement & Adaptation	153	31	CMG Development & Dissemination
CMG Development	116	27	CMG Development & Dissemination
CMG Utilisation by Staff	97	24	CMG applicability to patients, settings & resources
CMG Evidence Base & Research	162	23	CMG Development & Dissemination
Local/Rural Level	125	21	CMG Development & Dissemination
Social & Societal Issues	169	21	Patient Care & Standardisation
CMG Applicability to Setting	110	16	CMG applicability to patients, settings & resources
